# Data-driven design of orthogonal protein-protein interactions

**DOI:** 10.1126/scisignal.abm4484

**Published:** 2023-02-28

**Authors:** Duccio Malinverni, M. Madan Babu

**Affiliations:** 1 MRC Laboratory of Molecular Biology, Francis Crick Avenue, Cambridge Biomedical Campus, Cambridge CB2 0QH, UK; 2 Department of Structural Biology and Center of Excellence for Data Driven Discovery, St. Jude Children's Research Hospital, Memphis, TN 38105, USA

## Abstract

Engineering protein-protein interactions to generate new functions presents a challenge with great potential for many applications, ranging from therapeutics to synthetic biology. To avoid unwanted cross-talk with preexisting protein interaction networks in a cell, the specificity and selectivity of newly engineered proteins must be controlled. Here, we developed a computational strategy that mimics gene duplication and the divergence of preexisting interacting protein pairs to design new interactions. We used the bacterial PhoQ-PhoP two-component system as a model system to demonstrate the feasibility of this strategy and validated the approach with known experimental results. The designed protein pairs are predicted to exclusively interact with each other and to be insulated from potential cross-talk with their native partners. Thus, our approach enables exploration of uncharted regions of the protein sequence space and the design of new interacting protein pairs.

## Introduction

**Fig. 1:**
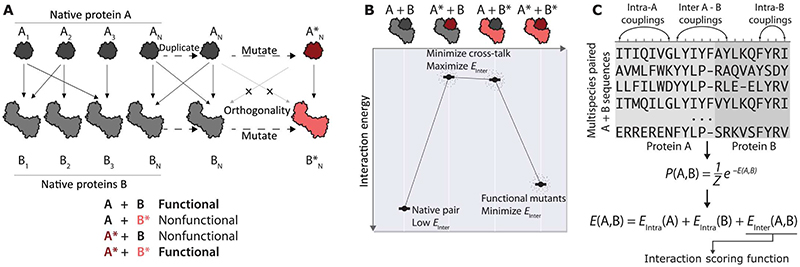
Generating new orthogonal interactors by simulating gene duplication and divergence. (**A**) Schematic of orthogonal PPI generation by duplication and divergence. A native interacting protein pair (A, B) is mutated to (A*, B*), ensuring that the cross-talk interactions A*-B and A-B* are avoided. Functional is defined as functionally insulated, without noncognate interactions. (**B**) Scoring function requirements. An effective scoring (or energy) function should score native pairs and mutant pairs favorably and assign unfavorable scores to cross-talk pairs. By convention, negative (low) interaction energy values indicate favorable interactions. (**C**) Coevolution-based statistical scoring function. A probabilistic model based on amino acid frequencies and cofrequencies observed in a multiple sequence alignment assigns a score *E*(A,B) to any pair of protein sequences. This score can be decomposed into intraprotein and interprotein terms representing the contributions from different sequence segments.

Protein-protein interactions (PPIs) are critical building blocks of diverse cellular functions. Cellular signaling relies on the ability of proteins to bind to their cognate interaction partners for efficient signal transduction upon stimulation. Interactions between proteins result in densely connected interaction networks within cells, within a tissue, and within an organism. To ensure that each member of a system interacts specifically with its target partners and that cross-talk is minimized, high interaction selectivity is required. In nature, novel functionality can emerge in these networks by the process of gene duplication and divergence, in which a preexisting gene is duplicated and then acquires mutations that result in the emergence of novel functions of the gene product through the generation of new interactions (1–3). Ensuring that the newly generated interactions are functionally insulated from those of their ancestors so that the newly duplicated gene products do not physically interact with their ancestral counterparts is vital in some instances (Fig. 1A) (4). Such functional insulation is referred to as an orthogonal interaction. Thus, the design of PPIs by repurposing preexisting proteins can generate engineered proteins that have potential for therapeutic and technological applications.

Designing such novel binding proteins relies on experimental approaches, such as directed-evolution techniques (including site-directed mutagenesis) (5, 6), and computational tools that use structural modeling (7–12), sequence analysis (13–15), or hybrid approaches (16). Although experimental selection procedures yield functional mutants with the desired properties, they incur high setup costs, are time consuming, and explore only relatively small portions of sequence spaces. Furthermore, their success depends on setting up experimental selection systems, which may be nontrivial to design for all molecular functions. In contrast, structurebased modeling enables the rapid exploration of mutants with relatively high accuracy; however, it requires a priori structural models of the interaction, is low throughput, and may not fully exploit all of the available sequence data. Structure-based approaches also assume a deep mechanistic understanding of the molecular processes needed to guide the choice of where to introduce mutations. Although these approaches have been successfully applied in several studies (7–12), their application to other systems and ability to efficiently explore large sequence spaces are intrinsically limited.

Here, we present a computational framework to design orthogonal PPIs by relying on the analysis of sequence data at the level of residue coevolution. Our approach mimics the natural phenomenon of duplication and divergence to create novel PPIs in cellular processes of interest. In contrast with previous coevolution-based methods that dissect the specificity of interacting proteins by analyzing the predicted effects of point mutations (13–15), we created a statistical model that we directly sampled to both generate and evaluate the interaction properties of a diverse set of candidate sequences with an arbitrary number of mutations in either interacting partner. We showed how this approach recapitulates experimentally determined orthogonal and promiscuous binding patterns of mutants. Furthermore, we present a strategy for the de novo design of proteins with specific binding properties (Fig. 1B).

## Results

### Capturing sequence covariation to score PPIs

The design of novel PPIs by computationally mutating interacting protein pairs *A* and *B* requires a scoring function that can quantify the likelihood of interaction. To build such a scoring function, we first quantified the patterns of sequence variation of known interacting proteins at the amino acid level (17, 18). For this, we first used remote homology search methods on publicly available databases (19, 20) to create a multiple sequence alignment (MSA) consisting of pairs of concatenated homolog sequences *A* and *B* (see Materials and Methods). Such coupled MSAs contain statistical signals observed throughout related species and encode the evolutionary relationships between amino acid positions located on the two interacting proteins. To concatenate pairs of interacting sequences, we leveraged prior knowledge of the PPI network. To construct such a paired alignment, we only required knowledge of interacting pairs without requiring knowledge of all noninteracting sequences (see Materials and Methods). If such prior knowledge is lacking, algorithmic matching strategies (21, 22) can be used to predict pairs of interacting sequences. We then used the MSA to train a global statistical model, **P** (*A*,*B*), that captured the intramolecular variation patterns and intermolecular covariation of the PPIs. The former captured the effects of variation on folding and biochemical function in isolation, and the latter captured information about the effect of variation on the likelihood of the interactions.

The statistical model **P**(A,B) has an associated energy function *E*(*A*,*B*) = −log P(*A*,*B*) (Fig. 1B). This can be factorized as *E*(*A*,*B*) = *E*_Intra_(*A*) + *E*_Intra_(*B*) + *E*_Inter_(*A*,*B*). The *E*_Inter_(*A*,*B*) term denotes the scoring function that we used to quantify the interaction likelihood of sequences with any number of nonsynonymous mutations away from the native sequences (Fig. 1C). A lower *E*_Inter_(*A*,*B*) score is indicative of a higher likelihood to interact. In this manner, we used the statistical model **P**(*A*,*B*) derived from known interacting pairs in naturally occurring sequences to computationally evaluate the effects of mutating the interacting protein pairs *A* and *B*.

### The scoring function discriminates the binding properties of mutated interacting protein pairs

**Fig. 2:**
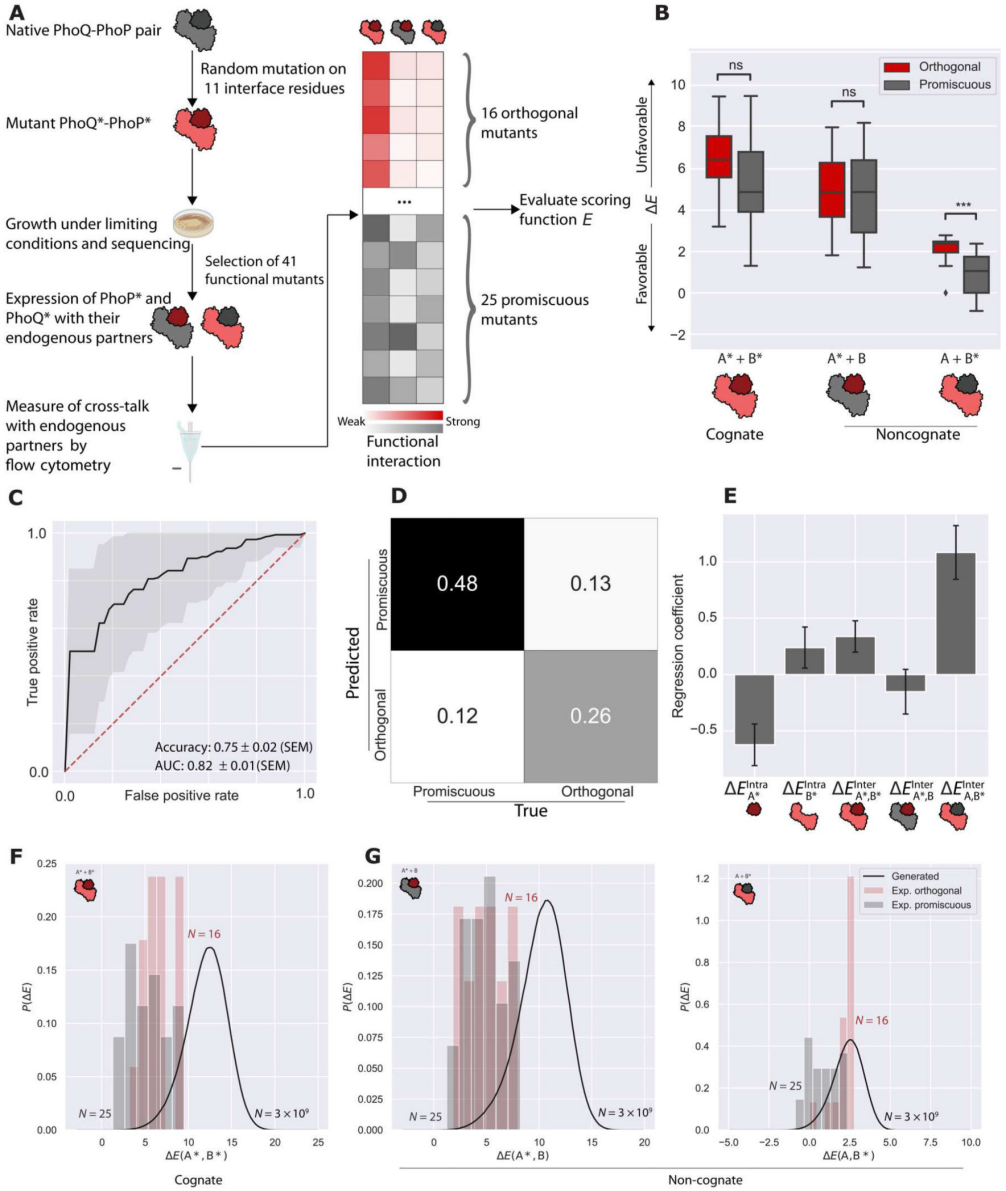
Characterization of the scoring function. (**A**) Overview of the experimental generation of the orthogonal and promiscuous mutants reported by McClune *et al*. (5). (**B**) Scoring of the 41 experimentally determined mutants (16 orthogonal pairs and 25 promiscuous mutants). All scores were normalized to the scores of the native proteins. Statistical significance tests were computed by two-sided Mann-Whitney tests with Bonferroni correction (***P < 0.001). The box plot center lines show the medians, box limits show the upper and lower quartiles, and the whiskers show the 1.5 interquartile range. Outliers are depicted by diamonds. (**C**) Receiver-operator characteristic (ROC) curve of the linear classifier using the statistical scores as input features. The black solid curve shows the average ROC curve. The shaded area corresponds to ±1 SD computed over 100 randomized cross-validations. (**D**) Confusion matrix of the linear classifier using the statistical scores as input features. Each entry represents the total fraction of mutants in a reference “true” group predicted to be in either of the two groups. The confusion matrix was averaged over 100 randomized cross-validations. (**E**) Average feature coefficients in the 100 logistic regression models. Larger regression coefficients (in absolute value) indicate features that contribute more to discriminating promiscuous from orthogonal mutant pairs. Error bars denote SDs over 100 randomized cross-validations. (**F** and **G**) Interaction scores (F, cognate; G, noncognate) of 3 × 10^9^ randomly generated mutants on the 11 experimentally determined positions (black curves), which were compared with the 41 experimentally determined functional mutants (histograms: red, 16 orthogonal pairs; gray, 25 promiscuous mutants).

To assess the performance of the scoring function, we first determined whether it could discriminate proteins with either promiscuous binding activity (multiple binding partners) or orthogonal binding activity (unique binding partners) from published experimental studies. We therefore analyzed a comprehensive dataset reported by McClune *et al*. (5) that consists of experimentally validated functional mutants of the bacterial PhoQ-PhoP two-component signaling system. The authors experimentally generated ~1 × 10^8^ variant PhoQ*-PhoP* (where the asterisk denotes a variant of the wild type) proteins by mutating 11 key interface positions. Their experimental selection system identified 41 pairs of mutant PhoQ*-PhoP* genes that were functional. Of these pairs, 16 were orthogonal interactors (that is, only the cognate PhoQ*-PhoP* interactions were observed), and 25 were promiscuous interactors (that is, in addition to the cognate PhoQ*-PhoP* interactions, either PhoQ*-PhoP or PhoQ-PhoP* noncognate interactions were observed) (Fig. 2A). To develop a PhoQ-PhoP scoring function, we created an MSA of ~24,000 PhoQ-PhoP homologous gene pairs from ~2200 species and trained a global statistical model on this MSA. The concatenated alignment contained 67 positions corresponding to PhoQ and 112 positions corresponding to PhoP. We then compared the predictions from the scoring function with those determined experimentally by McClune *et al*. (5).

For each of the 41 mutant pairs (PhoQ*-PhoP*, hereafter referred to as A*–B* for simplicity), we evaluated their interaction scores [Δ*E*_Inter_ = *E*(A*,B*) – *E*(*A*,*B*)], normalized with respect to the native pair, for their cognate interactions [Δ*E*_Inter_(A*,B*)] and noncognate interactions [Δ*E*_Inter_(A*,*B*) and Δ*E*_Inter_(*A*,B*)]. Comparing the normalized scores revealed the following trends (Fig. 2B). For the orthogonal and promiscuous pairs, the normalized scores of their cognate interactions Δ*E*_Inter_(A*,B*) were comparable (*P* = 0.26). This suggested that both the orthogonal and promiscuous pairs are functional when considering their cognate interactions. In addition, the normalized scores of the noncognate Δ*E*_Inter_(*A*,B*) interactions were significantly lower for the promiscuous pairs than for the orthogonal pairs (*P* = 2.4 × 10^-4^), indicating a stronger interaction likelihood for the promiscuous pairs. We observed no significant difference for the noncognate Δ*E*_Inter_(A*,*B*) interaction (*P* = 1). This finding suggested that our scoring function discriminates cognate from noncognate interactions.

Because of the different numbers of mutations in the two proteins, the three interaction scores [Δ*E*_Inter_(A*,B*), Δ*E*_Inter_(A*,*B*), and Δ*E*_Inter_(*A*,B*)] were not directly comparable. To explicitly quantify the performance of the scoring function, we built a linear classifier using the normalized components of the energy terms as input features for each mutant pair. The scoring function predicted the orthogonal or promiscuous nature of each of the 41 experimentally determined mutant pairs with a mean accuracy of 75% (mean area under the curve = 82%) (Fig. 2, C and D). Inspection of the regression weights of the classifier indicated that Δ*E*_Inter_(*A*,B*) contributed most to the discrimination between orthogonal and promiscuous pairs (Fig. 2E). Furthermore, the Δ*E*_Intra_(A*) term also strongly contributed to the discrimination, which indicates that whereas the model correctly identified the major discriminating features, the statistical decomposition into inter- and intraprotein components only approximated the underlying physical energetic contributions.

We assessed whether we could use the scoring function to select new orthogonal mutant sequences. We specifically tested whether the scoring function could replace the experimental selection procedure consisting of growth under limiting conditions and the selection step based on measuring cross-talk by flow cytometry (Fig. 2A) for the mutants in the same 11 positions in PhoQ-PhoP described by McClune *et al*. (5). To this end, we randomly generated 3 × 10^9^ mutant sequences in silico by fully randomizing those 11 positions. We computed the normalized interaction scores for the experimentally determined cognate (Fig. 2F) and noncognate (Fig. 2G) interactions and constructed the background random distribution of interaction scores for all 3 × 10^9^ mutant sequences. We compared the background random distribution of interaction scores for the in silico–generated mutants with the scores of the 41 experimentally determined mutants (Fig. 2, F and G) (5). The cognate interaction scores Δ*E*_Inter_(A*,B*) of the 16 experimentally determined functional mutants (that is, those with orthogonal interactions) were present in the lower end of the background random distribution (Fig. 2F). To corroborate this observation, we analyzed an additional set of observations composed of a PhoQ*-PhoP* combinatorial library generated by McClune *et al*. (5). In their study, 79 functional PhoQ*-PhoP* variants were expressed to build all of the resulting 79 × 79 possible variant combinations and functionally characterized. We classified these ~6200 variants into interacting and noninteracting pairs and compared their Δ*E*_Inter_(A*,B*) interaction scores on the basis of our evolutionary model (fig. S1). Although we observed a large overlap between the two distributions of the scores, the functional PhoQ*-PhoP* pairs displayed significantly lower Δ*E*_Inter_(A*,B*) scores compared with the nonfunctional ones (*P* = 0.00158). Together, these observations suggested that, to generate any potentially interacting mutants (that is, promiscuous, orthogonal, or both), we should select the sequences with lower cognate interaction scores Δ*E*_Inter_(A*,B*).

We then assessed orthogonality by comparing the noncognate interaction scores [Δ*E*_Inter_(A*,*B*) and Δ*E*_Inter_(*A*,B*)] of the experimentally determined mutants and for the in silico–generated mutants. The noncognate interaction scores Δ*E*_Inter_(*A*,B*) of the experimentally determined mutants displayed a bimodal distribution (Fig. 2G, right): The lower mode was enriched in promiscuous (nonfunctional) mutants and localized to the lower end of the background distribution, whereas the higher mode was enriched in orthogonal (functional) mutants and localized to the central region of the background distribution. In contrast, the other noncognate interaction scores of the experimental mutants Δ*E*_Inter_(A*,*B*) all localized to the lower tail of the background distribution and were thus not informative of orthogonality in this context (Fig. 2G, left). Comparing the noncognate scores Δ*E*_Inter_(A*,B*) and Δ*E*_Inter_(*A*,B*) highlighted an underlying asymmetry in the system, which also manifested as a strong correlation between the Δ*E*_Inter_(A*,B*) and Δ*E*_Inter_(A*,*B*) scores but a lack thereof between Δ*E*_Inter_(A*,B*) and Δ*E*_Inter_(*A*,B*) (fig. S2). Part of this asymmetry is likely attributed to the slightly higher number of promiscuous pairs in the experimental dataset that had *A*-B* noncognate interactions (12 of 25) compared with those that had A*-*B* noncognate interactions (7 of 25). Another six displayed interactions with both *A*-B* and A*-*B*. An additional likely contributing factor relates to a difference in evolutionary pressures acting on the two pairs of proteins, which can originate from a variable number of interaction partners and different structural constraints. Such differences in evolutionary rates between interacting proteins have been observed in the case of G protein–coupled receptor–G protein interactions (23) and in the TATA box–binding protein system (24). Varying protein lengths or interaction interface sizes (23), or both, can also result in variable evolutionary rates on different interacting partners. Together, the scoring function developed from the naturally occurring sequences predicted the experimentally determined binding properties (orthogonal or promiscuous) of mutant sequences that had never been seen in nature. Our findings also suggested that the scoring function may be a powerful tool for the design and discovery of new orthogonal mutant pairs.

### Computational strategy for de novo generation of orthogonal mutant interacting pairs

**Fig. 3:**
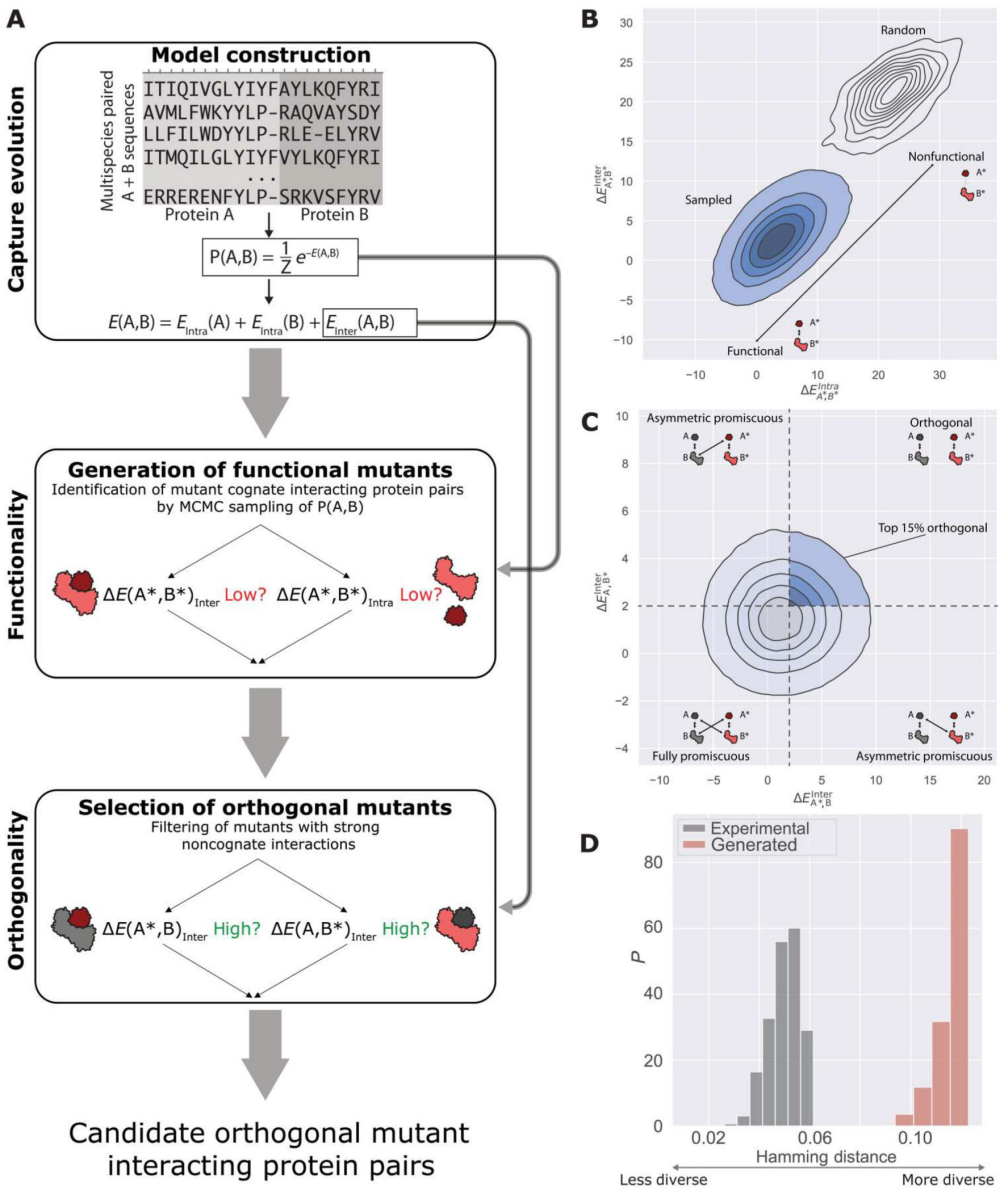
Generation of orthogonal sequences. (**A**) Schematic view of the computational design strategy. After building the statistical model and scoring function, we sampled the output of the model using MCMC modeling to generate a pool of cognate-interacting candidate mutants. In the second stage, sampled mutants were selected against noncognate interactions with the scoring function, retaining only mutants with unfavorable scores when evaluated with respect to their native partners. (**B**) First stage: Generation of cognate interacting mutants. The two histograms show the density plots for 5 × 10^5^ generated mutants, either by MCMC sampling (bottom left, sampled at *T* = 2.0) or by random sampling (top right density plot). (**C**) Second stage: Selection of orthogonal candidates. The 5 × 10^5^ mutants sampled by MCMC [mutants in the blue density plot in (B) were scored against their native partners to assess their likelihood of forming noncognate interactions]. The shaded region of the density plot shows the top 15% of mutants (~78,000 sequences) in the quadrant corresponding to orthogonal binding proteins. (**D**) Sequence diversity of the generated repertoire (red) and the 41 experimental mutants reported by McClune *et al*. (5) (gray). The depicted histograms show the distributions of all intragroup pairwise Hamming distances.

Given the performance of the scoring function, all possible variants may be computationally generated to identify mutants with specific binding properties. Although the sampling of all possible variants is feasible for small peptides and a small number of mutations, this can quickly become intractable for average-sized proteins. Because the statistical model captured the evolutionary constraints and tolerance for the mutations in the interacting proteins, the model captured information about which parts of the sequence spaces in the potential variants are likely to be functional in mediating an interaction. Therefore, this information can guide the choice of mutants that are most likely to be functional, reducing the size of the sequence space that needs to be sampled. On the basis of this principle, we designed a three-step strategy to generate novel candidate protein sequences that have orthogonal binding properties. First, we generated a large pool of potentially functional candidate mutants (cognate interacting pairs) with the statistical model. Second, we scored the generated mutants against their native interacting partners to assess their potential for noncognate interactions. Third, we selected the functional mutants (cognate interacting mutant pairs) with the lowest potential for noncognate interactions with the native sequences as candidate orthogonal interacting protein pairs (Fig. 3A).

To generate a large set of candidate functional mutants with a high likelihood of interaction, we used a Markov Chain Monte Carlo (MCMC) model to sample the sequences of pairs of interacting proteins from the statistical model **P**(A,B) by imposing a fixed number of mutations on each protein. Rather than uniformly sampling from an extensive background of the sequence spaces (for example, 20^11^ sequences for the PhoQ-PhoP system) and then selecting the sequences with low energy scores, the MCMC model directly samples the parts of the sequence spaces that are most likely to contain interacting protein pairs. Thus, the sequences returned by MCMC sampling naturally have a high likelihood of interaction according to our model based on low energy scores. We refer to this MCMC sampling based on low *E*(A,B) as the direct sampling method.

To control how closely (that is, the number of amino acid substitutions) around the native sequences we sampled, we introduced a virtual temperature (*T*) in the MCMC model. Using higher temperatures (*T* > 1) enabled sampling of more variable mutants but resulted in pairs with high overall energy *E*(*A*,*B*). Low temperatures (*T* < 1) enabled sampling of mutants close to the native sequences, resulting in mutants with low *E*(*A*,*B*) energies. For each of the sampled sequence pairs (A*,B*), we calculated the intramolecular [Δ*E*_Intra_(A*,B*) = Δ*E*_Intra_(A*) + Δ*E*_Intra_(B*)] and intermolecular Δ*E*_Inter_(A*,B*) energy terms. A previous study reported that generating sequences by MCMC to optimize enzymatic activity required the use of lower temperatures (*T* < 1) to sample functional variants (25). Here, the sampling temperature controlled both the functionality of the mutants and their potential for cross-talk. Thus, sampling conditions needed to be tuned outside of the natural value of *T* = 1. In addition, we compared the generated mutants with experimentally determined variants that were constrained to lie in a predetermined region of the sequence space because they were sampled from a fixed combination of positions. We do not, therefore, expect that these experimentally determined variants correspond to absolute low-energy states of the evolutionary model. Using higher sampling temperatures enabled the generation of variants covering a wider portion of the sequence space, encompassing and extending the previously small, experimentally explored region.

To find the most likely orthogonal sequences from these newly sampled sequences, we selected sequence pairs with high Δ*E*_Inter_ (A*,*B*) or Δ*E*_Inter_ (*A*,B*) because these had a low likelihood for noncognate interactions with the native sequences *A* and *B*. The observed asymmetric behavior between Δ*E*_Inter_ (A*,*B*) and Δ*E*_Inter_(*A*,B*) of the promiscuous and orthogonal mutants within the experimental set (Fig. 2G) appeared in the sampled sequence pairs (fig. S2). As indicated by the strong correlation between Δ*E*_Inter_(A*,B*) and Δ*E*_Inter_(A*,*B*) (fig. S2), the initial generation of variants having low Δ*E*_Inter_(A*,B*) automatically resulted in variants with lower Δ*E*_Inter_(A*,*B*), thus yielding candidate mutants in the relevant sequence space region. The second step, which consists of selecting the subset of variants with higher Δ*E*_Inter_(*A*, B*), is, in this case, a conservative criterion, by further requesting both crosstalk scores to be low.

We applied this strategy to investigate orthogonality in the PhoQ-PhoP system. We explored whether functional sequences could be identified in regions of the sequence spaces not previously explored. Starting from a set of ~1 × 10^10^ sequence pairs, we used direct sampling and obtained ~1 × 10^6^ independent mutant sequence pairs by introducing six mutations in PhoQ and five mutations in PhoP without restricting them to the interface. As expected, the sequences generated with direct sampling exhibited considerably better cognate interaction scores [lower Δ*E*_Intra_ (A*,B*) and Δ*E*_Inter_ (A*,B*)] than did 1 × 10^6^ completely random sequences (Fig. 3B). Sampling at *T* = 1 resulted in candidate mutants with better cognate interaction scores than those observed for the set of the 41 experimental functional mutants (fig. S3A). However, sampling at *T* = 1 yielded not only high cognate interaction scores but also high noncognate interaction scores for the mutants (fig. S3, B and C). To explore more diverse regions of the sequence spaces, we also sampled at higher temperatures (*T* = 1.2, 1.5, and 2), resulting in more variable candidate mutants (figs. S3 and S4). For subsequent analyses, we used the sequences generated at the higher virtual temperature (*T* = 2; Fig. 3B).

To select for orthogonality, we used the scoring function to evaluate the two noncognate interactions Δ*E*_Inter_ (A*,*B*) and Δ*E*_Inter_ (*A*,B*) of the generated mutants. Because orthogonality is defined as mutants with a low likelihood of forming noncognate interactions, we retained mutant pairs (A*,B*) with high Δ*E*_Inter_ (A*,*B*) and Δ*E*_Inter_ (*A*,B*) (Fig. 3C, top right quadrant) and selected the top 15% of candidate mutants in this region for further analysis (Fig. 3C; Materials and Methods). The top 15% of candidates resulted in a repertoire of ~78,000 candidate orthogonal mutants. The generated repertoire exhibited markedly higher variability (as measured by the pairwise Hamming distance between any two generated sequences) than did the set of 41 experimentally determined mutants (Fig. 3D). Therefore, our design strategy used the full statistical model **P**(*A,B*) to generate candidate functional mutants with a high level of repertoire diversity.

We further explored an alternative strategy to generate the initial candidate pool, which consists of a single step of sampling sequences favoring low Δ*E*_Inter_ (A*,B*) and Δ*E*_Intra_ (A*,B*) values, representing those with high functional potential, and jointly favoring high Δ*E*_Inter_ (A*,*B*) and Δ*E*_Inter_ (*A*,B*) values, representing those with low cross-talk potential. This was achieved with a loss function in the MCMC scheme, which includes the four ΔE terms and an additional temperature factor, T_2_, that controls the relative weighting between the original energy term and the cross-talk terms (see Materials and Methods). We refer to this strategy as the conditional sampling method. To test the generative capacities of this approach, we generated 1 × 10^5^ candidate mutants with this conditional sampling strategy for various *T*_2_ temperature values and compared the energy distributions of this repertoire with the distribution generated by direct sampling (fig. S5). The repertoire generated with conditional sampling was globally shifted toward sequences with higher noncognate energies Δ*E*_Inter_ (A*,*B*) and Δ*E*_Inter_ (*A*,B*), except for when *T*_2_ < 1. Increasing *T*_2_ primarily shifted the Δ*E*_Inter_(A*,*B*) toward higher values and had little effect on the Δ*E*_Inter_(*A*,B*) terms, representing a manifestation of the noncognate energy asymmetry that we observed for the experimental data. Whereas the cognate energies Δ*E*_Inter_(A*,B*) were affected by *T*_2_, the intramolecular energies Δ*E*_Intra_(A*,B*) were only modestly affected. Thus, the conditional sampling strategy efficiently sampled the variant sequence space in a tunable fashion achieved by the additional *T*_2_ parameter.

By comparing the energy distributions for the mutants generated at *T* = 1 and *T*_2_ = 1 with the energy values of the 41 experimentally determined mutants, we found that the mutants generated by the conditional sampling method still needed a filtering step to efficiently extract candidate mutants with likely orthogonal interactions (fig. S5). Therefore, we limited the complete conditional repertoire by selecting the 15% of mutants lying in the top right quadrant of the generated energy distribution (see Materials and Methods), as we did for the repertoire of mutants generated by the direct sampling method (Fig. 3C). We used principal components analysis (PCA) to compare this filtered set of mutants generated with conditional sampling with the filtered set generated with direct sampling (fig. S6). Both methods yielded variant sequences exploring mostly overlapping regions of the sequence space. The analysis of these two approaches showed that the direct sampling approach requires a unique sampling temperature *T* to control both the functionality of the mutants and their cross-talk interactions, which partially explains the need to use a nonstandard temperature, *T* = 2. In contrast, the conditional sampling strategy introduces an additional parameter that controls the cross-talk interactions. Both strategies required additional filtering. However, both sampling strategies yielded similar mutant repertoires; therefore, we focused on characterizing the repertoire generated with the direct sampling approach.

### Characterization of a de novo–generated repertoire of candidate orthogonal mutants

**Fig. 4:**
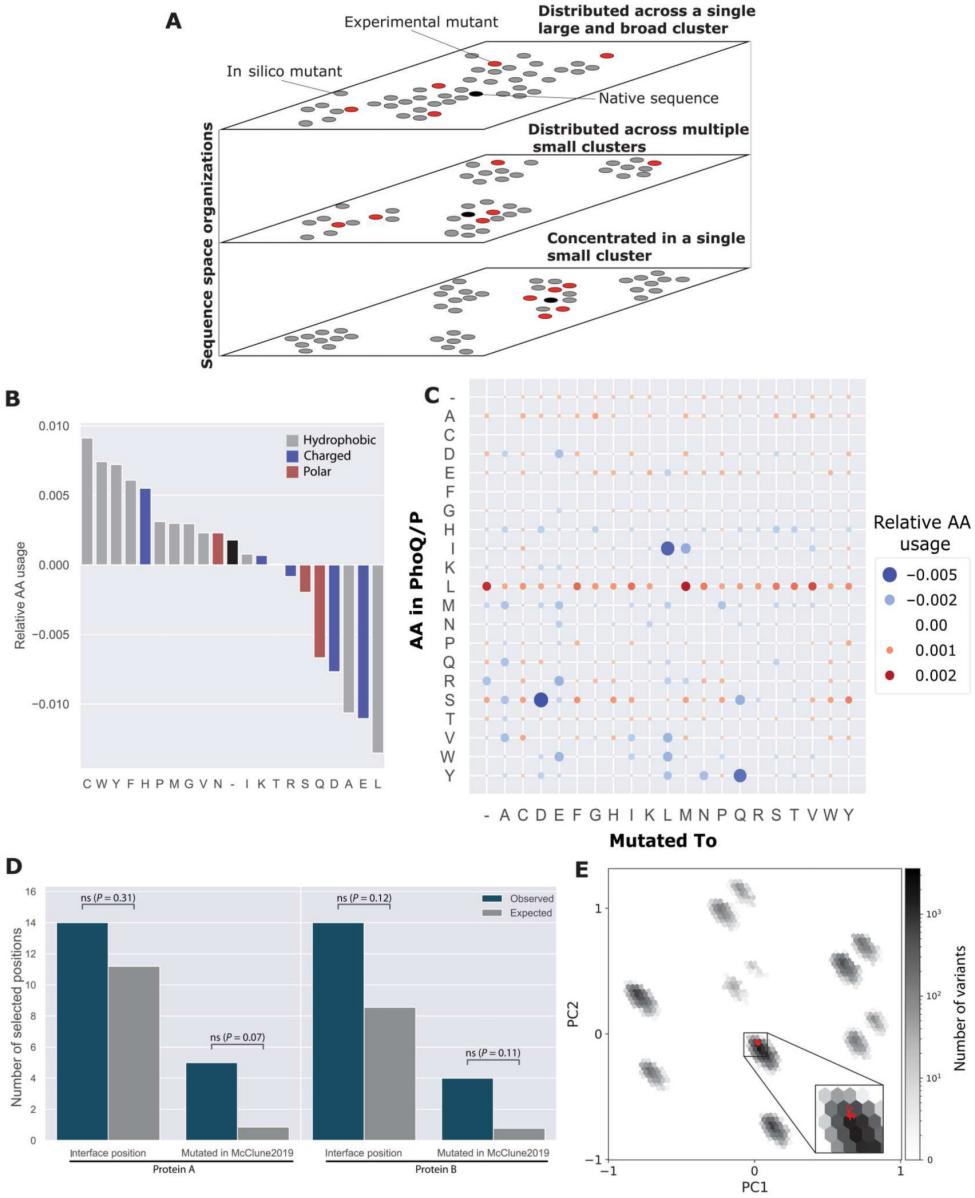
Characterization of the repertoire of the generated orthogonal sequences. (**A**) Schematic view of various possible organizations of the mutant sequence spaces. Gray dots represent sampled mutants, whereas red dots represent experimentally determined mutants. Black dots represent the native, wild-type sequence. (**B**) Relativeamino acid usage in the ~78,000 generated orthogonal mutants, normalized by the amino acid usages in the 5 × 10^5^ variants sampled by MCMC before selection for orthogonality. (**C**) Amino acid substitution enrichment analysis. Dots in the matrix represent the relative frequency that an amino acid in PhoQ or PhoP (*y* axis) was mutated to a particular amino acid (*x* axis) in the ~78,000 generated orthogonal variants, normalized by the same quantity measured in the 5 × 10^5^ variants sampled by MCMC before selection for orthogonality. (**D**) Enrichment analysis of the interface positions and experimentally mutated positions in the ~78,000 generated orthogonal variants. Statistical significance was assessed by two-tailed Fisher exact tests. (**E**) PCA of the mutant sequence spaces. The plot shows the two-dimensional density of the selected orthogonal candidate mutants. Red dots represent the 41 experimentally interacting mutants (both orthogonal and promiscuous).

We sought to understand the characteristics of the broad sequence variability in the candidate orthogonal mutant repertoire generated by direct sampling (Fig. 3D), both in terms of sequence composition and mutated positions. Therefore, we tested whether the ~78,000 generated mutants contained mutations beyond the structural interface and the 11 mutated positions reported by McClune *et al*. (5) and whether the mutants formed distinct sequence clusters. We also compared how the experimentally generated mutants and the candidate orthogonal mutant repertoire existed within the sequence space (Fig. 4A). This analysis can indicate the different ways a system may achieve orthogonality. A single broad cluster of sequences indicates one principal way to mutate the system to achieve orthogonality. In contrast, the presence of multiple clusters of sequences may indicate that multiple different solutions can result in orthogonality.

We first analyzed the amino acid compositions of the generated repertoire. Comparing the amino acid usage of the filtered repertoire with those of all of the MCMC-generated mutants showed that there was a slight enrichment in the use of hydrophobic mutations in the candidate orthogonal mutant repertoire (Fig. 4B). However, in absolute numbers, all amino acids were present in the repertoire (fig. S7), indicating that the ~78,000 mutants do not rely on a single biochemical property, such as hydrophobicity or charge, to achieve orthogonality. Instead, the sampling and filtering strategy modulated both properties, with a slight preference for hydrophobicity. Assuming that hydrophobic mutations represent a proxy for negative design and that charged mutations predominantly represent positive design (26, 27), these results suggested that the evolutionary-based design of orthogonal interactions leverages both types of design, with a stronger element of negative design against interactions with endogenous partners to achieve orthogonality. Detailed analysis of amino acid substitutions (Fig. 4C) showed that some amino acids were likely substituted without a strong preference for the substitution (for example, leucine), whereas other substitutions were selected against (for example, serine-to-aspartate or isoleucine-to-leucine). Such specific enrichments in amino acid substitutions likely go beyond simple physicochemical properties and are likely a consequence of precise molecular mechanisms involving the properties of specific residues.

We also analyzed whether the positions mutated in the generated repertoire exhibited enrichment for positions in the interaction interface or for the 11 experimentally selected positions (5). We observed only weak enrichment of the interface positions (fig. S8, A and B) and of the experimentally mutated positions on both proteins (Fig. 4D and fig. S8, C and D). Hence, the positions mutated to achieve the candidate orthogonal interactions extended beyond the interface and were located throughout the interacting proteins (fig. S8D). Furthermore, pairs of positions mutated together (cooccurring pairs) were not enriched in interface contacts when assessed by multiple methods (fig. S9, A to C). These observations suggested that mutations in positions away from the interface likely contribute to orthogonality, possibly through allosteric mechanisms.

We characterized the sequence space distribution in the generated and filtered repertoire by PCA. Visualization of the sequence spaces revealed that the repertoire was composed of at least 12 clusters of mutants (fig. S10A) with the number of mutants in each cluster varying by two orders of magnitude (fig. S10B). Analysis of the cluster similarities did not show any strong hierarchical organization of the clusters (fig. S10C). The clusters contained specific mutations (figs. S11 to S13), in terms of not only the frequency of the substituted amino acids (figs. S11 and S12) but also the positions of the amino acids (fig. S13). Many of the frequently mutated positions were mapped to residues away from the interface (fig. S13). Furthermore, the 41 experimentally identified mutants all localized to the most populated cluster (Fig. 4E and fig. S10, cluster 5).

To test the potential origins of the clustered variant sequence space and the location of the 41 experimental mutants, we generated a random sample of variants by MCMC sampling, limiting the mutated positions at the same 11 positions as used in the study by McClune *et al*. (5). Similar to the 41 experimental variants, all mutants of this constrained repertoire localized in cluster 5, showing that the position restraints limit the available sequence space explored by the experimental procedure (fig. S14A). To investigate the origins of the other clusters, we generated 100 sampling simulations in which we fixed 11 randomly selected positions in each simulation. Fixing the mutable positions generally resulted in all of the mutants falling in one or two distinct clusters per simulation, with most present in cluster 5 (fig. S14B). We observed that certain random choices of mutable positions resulted in generated variants localizing to other clusters (fig. S14, C to F). Inspection of these specific random choices showed that the selected mutable positions generally contained positions that were often mutated in our original repertoire (figs. S11 and S12). Thus, we concluded that the clustered structure of the sequence space was predominantly a consequence of the use of a combination of specific positions in the generative process. The various identified clusters mostly used a combination of similar positions, with a modulation of the exact amino acid usage at these residues (figs. S11 to S13). Thus, the global structure of the mutants' sequence space is an interplay between which precise positions are mutated and which amino acids populate them in the various clusters. Together, these analyses highlight how the proposed design strategy can effectively explore broad sequence spaces around native sequence pairs.

## Discussion

The methodology presented here and the accompanying software enable the efficient generation of novel candidate mutants with binding properties orthogonal to an endogenous interaction network. Our results showed that the coevolution-based scoring function can effectively discriminate orthogonal from promiscuous mutants and that we can efficiently sample it as a generative model. Training the model requires hours to days, depending on the alignment size (in terms of the number of amino acid positions). With our current implementation, we trained and refined the PhoQ-PhoP model in 3 days. Once trained, exploring millions to billions of candidate mutants can be performed in a matter of minutes to a few hours, and exploring different sampling regimes (that is, varying the repertoire diversity by tuning the sampling temperature *T*) can be rapidly performed. With the PhoQ-PhoP system, generating ~70,000 orthogonal candidate sequences required ~18 hours with six central processing unit cores. We can thus rapidly generate a large and diversified repertoire of candidate mutants for further analyses. Furthermore, this is possible whether or not structural information of the interacting proteins is available.

The ability to generate orthogonal sequences to interacting proteins relies on a key assumption—that the frequency of mutations observed in the sequences used to train the model reflects their effects on PPIs. Therefore, it is important to choose the right sequences at the right level of divergence when generating the model. Nevertheless, distantly related sequences can provide potentially new solutions for achieving orthogonality because divergent homologs of interacting proteins can use different interfaces (28, 29). Thus, a balance between using divergent yet sufficiently similar sequences may yield an optimal training set.

Furthermore, the observation that the generated repertoire consists of mutations both in the interaction interface and distal sites suggests that evolutionary-based interaction models capture multiple types of effects that tune PPIs. In particular, the presence of mutations far away from the interaction interface might suggest that the introduced mutations can act as allosteric modulators or indirectly tune the binding properties by tuning protein stability, dynamics, or entropy. Such long-distance effects on protein-protein and protein-DNA interactions through various mechanisms have been reported in several cases, such as in noninterface positions in the bacterial ParD-ParE toxin–anti-toxin system (30), modulation of DNA binding of the complement-reactive protein transcription factor by distal positions (31, 32), and the control of oligomeric states of PyR proteins by noninterface positions affecting the protein dynamics (33).

Multiple critical biomedical applications can benefit from the ability to generate a diverse repertoire of novel binding proteins, including the design of peptide inhibitors for cellular receptors involved in human diseases. Furthermore, designing orthogonal receptor-ligand systems may permit the engineering of novel biochemical tools to precisely investigate cellular signaling in model organisms by introducing biochemical control mechanisms to modulate downstream steps. The proposed methodology will benefit from the continuous growth of genomic data that are rapidly becoming available. Specifically, the advent of sequencing initiatives, such as the Darwin Tree of Life Project, which will sequence the genomes of ~70,000 eukaryotic species, will greatly improve the prediction quality of eukaryotic-specific systems. Studies of eukaryotic systems will be accompanied by methodological challenges, in particular, the necessity to match interacting paralogs when building the paired sequence alignment. With a higher number of paralogs compared with prokaryotic systems, subcellular compartmentalization, tissue-specific gene expression profiles, and more complex regulatory mechanisms, the matching of interacting sequence pairs may become a challenge for eukaryotic systems, and coevolution-based methods, such as IPA (21) or PPM (22), can be leveraged to iteratively build near-optimal paired alignments. In addition, advances in protein-language modeling are paving the way for methods that implicitly learn matching strategies in a semi-unsupervised fashion (34, 35), whereas advances in deep mutational scanning approaches enable the incorporation of variation data in the generative process. This has the potential to continually improve the performance of the proposed methodology (36) because deep mutagenesis datasets for both tested interacting proteins will facilitate nuanced validation and fine-tuning. The scheme proposed here has the flexibility to incorporate such validation data and can be further complemented with advances in deep learning–based modeling of protein sequence spaces (37–44). The large sizes attained by sequence databases will lead to continuous performance improvements and so will further use of data-intensive, state-of-the-art, deep-learning architectures (40, 45) for modeling sequence spaces. Harnessing the power of information in both the sequences derived from millions of years of evolution (that is, natural sequences) and the technological advances over the last few years (for example, deep mutational scanning) will profoundly affect how new protein interactions are designed. We therefore envision that the generative modeling scheme proposed here will act as a methodological scaffold for future computational and experimental design of orthogonal PPIs.

## Materials And Methods

### Construction of the concatenated MSA

To build a concatenated alignment of the histidine kinase (HK)–response regulator (RR) bacterial two-component system, we downloaded all of the deposited prokaryotic two-component systems for 3198 bacteria from the Prokaryotic 2-Component Systems (P2CS) database (46). For each organism, we extracted all of the HK sequences that belonged to the PFAM (47) HisKA family (PF005512) and the RR sequences that belonged to the PFAM ResReg family (PF000072) by performing homology searches with the hmmsearch tool from hmmer software v3.3.0 (48) with standard inclusion thresholds. We further filtered out any unorthodox kinases (often containing a kinase and regulator fusion product) by retaining only the kinase sequences annotated as classic HKs in P2CS. Each HK (and its respective RR) sequence was then aligned to their respective PFAM hmm models with the hmmalign tool from the hmmer software suite. Cognate HK-RRs were then matched for each organism by pairing HK and RR sequences whenever they were found in the same operon (that is, on the same strand and separated by fewer than 200 bp). This procedure resulted in a total of 23,933 paired sequences that were distributed among 2227 species. To reduce redundancy and limit the sampling bias due to overrepresented organisms in the sequence databases, we filtered our matched alignment by retaining sequence pairs having a maximum of 90% pairwise sequence identity [filtering performed with the hmmfilter utility of the hmmer software (48) resulted in a redundancy-filtered MSA containing 10,565 paired HK-RR sequences]. Note that because of the unsupervised nature of the statistical model used, we only required knowledge of truly interacting pairs to build the concatenated alignment, without explicit knowledge of any negative interactions. This is particularly important because it is experimentally easier to determine positive PPIs, whereas identifying noninteracting protein pairs is generally more challenging. In addition, the use of coevolutionary-based models to dissect interaction specificity at the paralog level can effectively recover interactions even between pairs missing from the training set (13). This highlights the importance of having high confidence in pairing interacting partners while also underlying the robustness of the approach to missing data. In particular, the operon structure of interacting paralogs is a highly efficient way to build such paired alignments (21, 22, 49).

### Inference of the global statistical model

We modeled the mutational landscape of the paired MSA by the global statistical Potts model over the protein sequence spaces consistent with the first and second moments (17, 18, 25):

(1) $$P(s)=\frac{1}{Z}\exp(\sum_{i=1}^{N}h_{i}(s_{i})+\sum_{i,j=1}^{N,N}J_{ij}(s_{i},s_{j}))\equiv \frac{1}{Z}\exp(-E(s))$$

In this formula, ***s*** = (*s*_1_, *s_2_*, …, *s_N_*) denotes a protein sequence, and each position *s_i_* can span 21 values (20 amino acids and 1 gap state). To learn the model parameters from the data in an MSA, we used a Boltzmann learning scheme (50). This explicitly minimized the negative log likelihood of Eq. 1 evaluated on the sequence data by gradient descent. To avoid overfitting and divergences due to combinations of amino acids that did not occur in the data, an *L_2_*-regularized, negative log likelihood of Eq. 1 was instead maximized

(2) $$L_{\text{Reg}}(\theta )=L(\theta )+\lambda \sum_{i=1}^{N}\sum_{A=1}^{21}h_{i}{\left(A\right)}^{2}+\lambda \sum_{i,j=1}^{N}\sum_{A,B=1}^{21}J_{ij}{\left(A,B\right)}^{2}$$

where θ denotes the model parameters {*h_i_*, *J_ij_*}, the negative log likelihood of the data is given by

(3) $$L(\theta )=-\sum_{b=1}^{B}\log P_{\theta }(s_{b})$$

and the summation is taken over the *B* sequences in the MSA. The regularized log likelihood was approximatively minimized by the standard gradient descent

(4) $$\theta_{i+1}=\theta_{i}-\eta \nabla L_{Reg}(\theta_{i})$$

and we used a step size of η = 0.01. At each iteration, the gradient ∇*L*_Reg_(θ*_i_*) was estimated by MCMC sampling of the Boltzmann distribution Eq. 1, with the parameters θ_*i*_ of the current iteration (50). We performed the sampling by running 128 parallel MCMC samplings and pooling all of the sampled sequences to estimate the gradient. Each parallel MCMC sampling consisted of 100,000 sweeps, recording a configuration every 100 sweeps. A sweep was formed by *N* Monte Carlo steps, where *N* was the number of amino acid positions in the MSA. For a Monte Carlo step, we randomly selected a residue and mutated it to any of the 20 other possible states, accepting the move by the Metropolis criterion. We performed 2000 gradient descent steps to train the model. After the training phase was finished, the model parameters were shifted to the zero-sum gauge (51) by imposing

(5) $$\sum_{A=1}^{21}h_{i}(A)=0\forall i$$

and

(6) $$\sum_{A,B=1}^{21}J_{ij}(A,B)=0\forall i,j$$

To verify that the reported findings of this study do not depend strongly on these parameters, we trained an additional model for 15,000 training steps using 500 sweeps between sample recordings. The evaluation of the statistical energies on the experimentally determined orthogonal and promiscuous variants revealed results that were consistent with a model trained for less time, showing the robustness of the results of these choices (fig. S15).

### Scoring of pairs of interacting sequences

To score pairs of sequences, we considered the statistical energy term in Eq. 1

(7) $$E(s)=\sum_{i=1}^{N}h_{i}(s_{i})+\sum_{i,j=1}^{N,N}J_{ij}(s_{i},s_{j})$$

Because of the sign convention chosen in Eq. 1, negative scores for *E*(***s***) corresponded to more favorable sequences. For the two sequences **A** = (*a*_1_, *a*_2_, …, *a_N_A__*) and **B** = (*b*_1_, *b*_2_, …, *b_N_B__*), the concatenated sequence is given by ***s*** = (*a*_1_, *a*_2_, …, *a_N_A__*, *b*_1_, *b*_2_, …, *b_N_B__*), and the score (Eq. 7) can be decomposed as

(8) $$E(s)=\sum_{i=1}^{N_{A}}h_{i}(s_{i})+\sum_{i=N_{A}+1}^{N_{A}+N_{B}}h_{i}(s_{i})+\sum_{i=1,j=1}^{N_{A},N_{A}}J_{ij}(s_{i},s_{j})+\sum_{i=N_{A}+1,j=N_{A}+1}^{N_{A}+N_{B},N_{A}+N_{B}}J_{ij}(s_{i},s_{j})+\sum_{i=1,j=N_{A}+1}^{N_{A},N_{A}+N_{B}}J_{ij}(s_{i},s_{j})=\underset{E_{\text{Intra}\;}(A)}{\underset{︸}{\sum_{i=1}^{N_{A}}h_{i}(s_{i})+\sum_{i=1,j=1}^{N_{A},N_{A}}J_{ij}(s_{i},s_{j})}}+\underset{E_{\text{Intra}\;}(B)}{\underset{︸}{\sum_{i=N_{A}+1}^{N_{A}+N_{B}}h_{i}(s_{i})+\sum_{i=N_{A}+1,j=N_{A}+1}^{N_{A}+N_{B},N_{A}+N_{B}}J_{ij}(s_{i},s_{j})}}+\underset{E_{\text{Inter}\;}(A,B)}{\underset{︸}{\sum_{i=1,j=N_{A}+1}^{N_{A},N_{A}+N_{B}}J_{ij}(s_{i},s_{j})}}=E_{\text{Intra}\;}(A)+E_{\text{Intra}\;}(B)+E_{\text{Inter}\;}(A,B)$$

Therefore, the score of each pair of concatenated sequences can be decomposed into two intraprotein contributions, *E*_Intra_(*A*) and *E*_Intra_(*B*), and an interprotein contribution, *E*_Inter_(*A*, *B*), according to (Eq. 8). When considering the scores of mutants A* and B*, we normalized them with respect to the scores of the concatenated native proteins, that is,

(9) $$\Delta E(A^{*},B^{*})=E(A^{*},B^{*})-E(A,B)$$

and similarly for *E*_Intra_ and *E*_Inter_. Last, cross-talk scores were obtained by evaluating Δ*E*_Inter_ on the concatenation of a mutant protein with its native partner, that is, Δ*E*_Inter_(A*, *B*) or Δ*E*_Inter_(*A*,B*). To check how the decomposed scores Δ*E*_Intra_(*A*) and Δ*E*_Intra_(*B*) behave when compared with their equivalent measures computed on independent models, we trained two models using only the parts of the MSA corresponding to PhoQ (MSA_A, 67 positions) and to PhoP (MSA_B, 112 positions). Comparing the Δ*E*_Intra_(*A*) and Δ*E*_Intra_(*B*) components of the 41 experimentally determined variants showed that the intraprotein components of the energies were strongly correlated between the joint and independent models (fig. S16). We observed that the correlation was significantly stronger for the Δ*E*_Intra_(PhoP) energies, with Pearson correlation coefficients of 0.85 versus 0.98. This is potentially due to the PhoQ sequences in the joint alignments being shorter than the PhoP sequences (*L*_PhoQ_ = 67, *L*_PhoP_ = 112) and potentially having different evolutionary rates [see, for example, the study by Ovchinnikov *et al*. (52)].

### Classification of experimental orthogonal and promiscuous partners by logistic regression

For each of the 41 experimentally determined mutant pairs (A*,B*), we built a feature **x** vector composed of five entries

(10) $$x(A^{*},B^{*})=\;\text{[}\Delta E_{\text{Intra}\;}(A^{*}),\Delta E_{\text{Intra}\;}(B^{*}),\Delta E_{\text{Inter}\;}(A^{*},B^{*}),\Delta E_{\text{Inter}\;}(A^{*},B)\times \Delta E_{\text{Intra}\;}(A,B^{*})]$$

These feature vectors were then used to train a logistic regression-based classifier with a default regularization constant *C* =1. The training was performed on a random split of the dataset, consisting of a training set of 31 training samples and a test set of 10 samples. We repeated the random split and training 100 times and reported the performance metrics (that is, model accuracy, area under the curve, and confusion matrix) as averages computed over the randomly split 100 test sets. All supervised training was performed with the sklearn Python library (53).

### De novo generation of orthogonal protein pairs by direct sampling

To generate new candidate mutants for further evaluation, we sampled the distribution Eq. 1 by MCMC. To control the variability of the generated mutants, we introduced a virtual temperature factor in Eq. 1, effectively sampling from

(11) $$P(s)=\frac{1}{Z}\exp(\frac{-\sum_{i=1}^{N}h_{i}(s_{i})-\sum_{i,j=1}^{N,N}J_{ij}(s_{i},s_{j})}{T})$$

We performed the sampling with an in-house written code called OIS (Orthogonal Interacting Sequences), sampling a total of 5 × 10^5^ mutants composed of six mutations in the PhoQ protein and five in the PhoP protein. Each mutant was saved after 100 sweeps (as described earlier), and its Δ*E*_Inter_(A*, B*) and Δ*E*_Intra_ = Δ*E*_Intra_(A*) + Δ*E*_Intra_(B*) scores were computed. For PhoQ-PhoP, we found that using a virtual temperature of *T* = 2 yielded satisfactory results. The natural temperature *T* = 1 resulted in mutants with not only favorable functional scores [that is, low Δ*E*_Inter_(A*, B*) and Δ*E*_Intra_ values] but also favorable cross-talk scores. Each sampled mutant was then decomposed into its two composing proteins, A* and B*, and paired with its native partner to evaluate the cross-talk scores Δ*E*_Inter_(A*, *B*) or Δ*E*_Inter_(*A*, B*). Last, mutants with high noncognate interaction scores Δ*E*_Inter_(A*, *B*) and Δ*E*_Inter_(*A*, B*) (that is, with a low cross-talk probability) were selected to generate the final synthetic orthogonal repertoire. To perform the last selection step, we imposed a fraction of mutants **F** to be selected from the upper-right quadrant in the Δ*E*_Inter_(A*, *B*) versus Δ*E*_Inter_(*A*, B*) plot (Fig. 3C). To this end, we scanned the line equally dissecting the quadrant, which passed through the mean of the distribution, for a point defining the two threshold values (*E*_1_ and *E*_2_) to achieve a fraction **F** of the mutants with *E*_1_ <Δ*E*_Inter_(A*, *B*) and *E*_2_ < Δ*E*_Inter_(*A*, B*). All mutants falling above the two thresholds were then selected as final candidate mutants.

### De novo generation of orthogonal protein pairs by conditional sampling

An alternative sampling strategy, called conditional sampling, consists of sampling from a modified loss function that favors variants with low cognate interaction scores and intramolecular statistical energies and jointly favors variants with high noncognate interactions. To implement this scheme, we generated variants with the conditional sampling strategy by sampling from the following distribution:

(12) $$\begin{array}{r} P(s)=\frac{1}{\tilde{Z}}\exp(\frac{-E_{\text{Intra}\;}(A^{*})-E_{\text{Intra}\;}(B^{*})-E_{\text{Inter}\;}(A^{*},B^{*})}{T}\\ +\frac{E_{\text{Inter}\;}(A^{*},B)+E_{\text{Inter}\;}(A,B^{*})}{T_{2}}) \end{array}$$

where the energy terms were defined in Eqs. 7 to 9. The virtual temperature *T*_2_ controls the relative weighting of the cognate versus noncognate energy scores in the total loss function. Samples were generated with the same MCMC scheme as for the direct sampling case, using the loss function defined by Eq. 12 instead of Eq. 11. Similarly, as for the direct sampling strategy, candidate variants were filtered after having been generated by selecting a fraction **F** of mutants to be selected from the upper-right quadrant in the Δ*E*_Inter_(A*, B) versus Δ*E*_Inter_(A, B*) plane. We found in practice that using this scheme with the “natural temperatures” *T* = 1 and *T*_2_ = 1 yielded repertoires very similar to those obtained by direct sampling with *T* = 2.

### Analysis of the set of in silico–generated orthogonal protein pairs

We considered a homologous two-component system to determine the interface positions in the PhoQ-PhoP complex (Protein Data Bank ID: 3DGE). Interface positions were defined as residues with an interprotein distance between heavy atoms of <6 Å in the crystal structure. The 11 positions experimentally mutated in the study by McClune *et al*. (5) all localized to this interface definition and corresponded to position indexes 18, 21, 22, 25, 38, and 39 for PhoQ and 76, 78, 79, 82, and 83 for PhoP in the concatenated PhoQ-PhoP alignment. PCA of the sequence space of the generated mutants was performed with the dcaTools package (54). The 12 clusters were identified by visual inspection and are consistent with k-means clustering. The hierarchical clustering of the clusters (fig. S10) was performed with the sklearn library (53) with single linkage and the Euclidean distance between the mutation profiles (figs. S11 and S12) of each cluster.

### Sequence logos

The sequence logos depicted in figs. S11 and S12 were computed with the Logomaker python library (55). The logos represented the amino acid frequencies of the mutations at each position; that is, the amino acids of the native sequences were not depicted. Thus, the total height of the stacked symbols at each position was proportional to the mutation frequency of the position.

## Supplementary Material

Figs S1 to S16

MDAR Reproducibility Checklist

## Data Availability

The MSA used to perform the PhoQ-PhoP analysis is openly available on the github repository of the OIS software at https://github.com/mb-group/ois. The code used to perform the proposed method is available at https://github.com/mb-group/ois under a General Public License (GPL-3). The repository contains the full code base and an accompanying tutorial for generating orthogonal binding proteins. The code is also available at Zenodo (https://doi.org/10.5281/zenodo.7636981). All data needed to evaluate the conclusions in the paper are present in the paper or the Supplementary Materials.
