# Supplementary material for: Data-driven design of orthogonal protein-protein interactions
Source: Sci Signal. Author manuscript; Available in PMC 2023 May 24. (PMC7614567; doi:10.1126/scisignal.abm4484)
Supplement: Figs S1 to S16 [file EMS175605-supplement-Figs_S1_to_S16.pdf]

Science Signaling
AAAS

Supplementary Materials for

Data-driven design of orthogonal protein-protein interactions

Duccio Malinverni and M. Madan Babu

Corresponding author: Duccio Malinverni, duccio.malinverni@stjude.org;
M. Madan Babu, madan.babu@stjude.org

Sci. Signal. **16**, eabm4484 (2023)
DOI: 10.1126/scisignal.abm4484

The PDF file includes:

Figs. S1 to S16

Other Supplementary Material for this manuscript includes the following:

MDAR Reproducibility Checklist

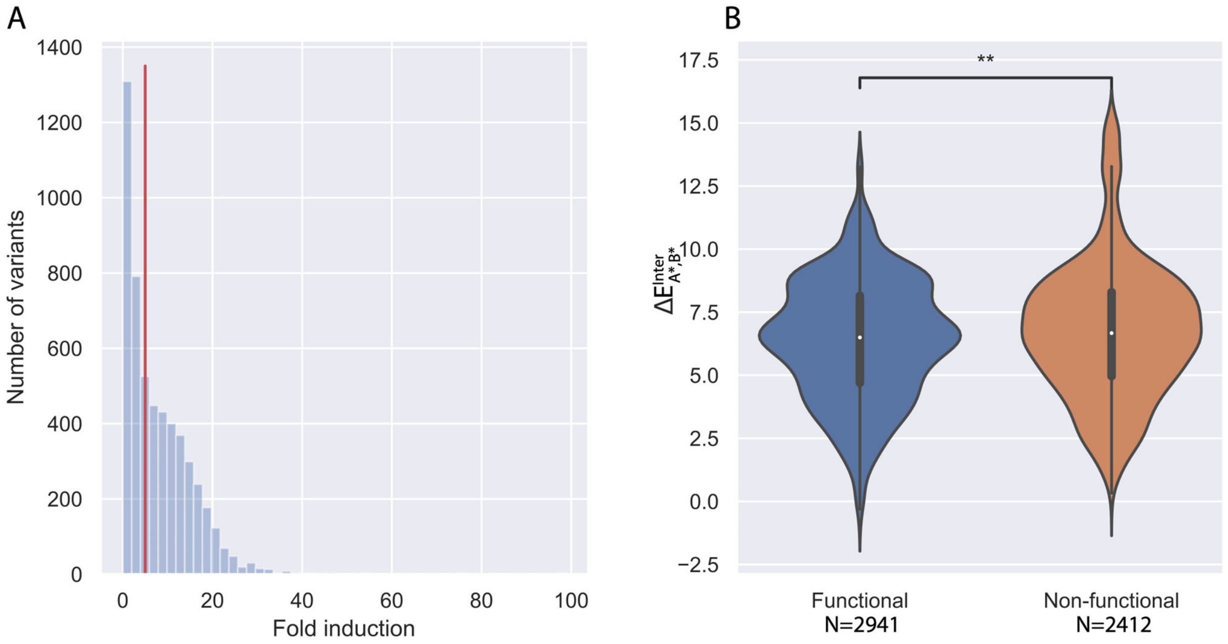

Fig. S1. Discriminatory analysis of a 79 × 79 combinatorial PhoQ*-PhoP* mutant library. (**A**) Distribution of experimentally determined fold-induction of the 79 × 79 PhoQ*-PhoP* variants. Higher fold-induction indicates interacting variants [see Materials and Methods and McClune *et al*. (*5*) for the exact definition of variant fold-induction]. The vertical red line indicates the threshold separating nonfunctional from functional variants. (**B**) Comparison of the ΔE_{Inter} scores for all of the variants in the two groups. ** significance: $P < 0.01$ threshold; $P = 0.00158$ by Mann-Whitney test with Bonferroni correction.

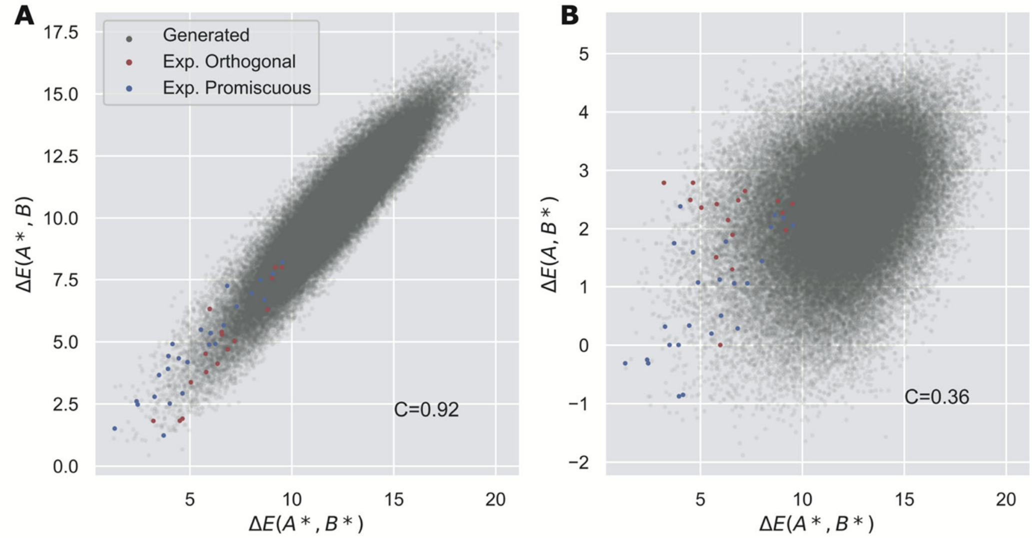

Fig. S2. Correlation between cognate and non-cognate normalized interaction scores. (**A** and **B**) The interaction scores were computed for both the 3×10^9 randomly generated mutants (gray) and the 41 experimentally determined mutants (red and blue). C denotes the Pearson correlation coefficient. (A) Correlation between cognate interaction energies $\Delta E_{Inter}(A^*,B^*)$ and noncognate interaction energies $\Delta E_{Inter}(A^*,B)$. (B) Correlation between cognate interaction energies $\Delta E_{Inter}(A^*,B^*)$ and noncognate interaction energies $\Delta E_{Inter}(A,B^*)$.

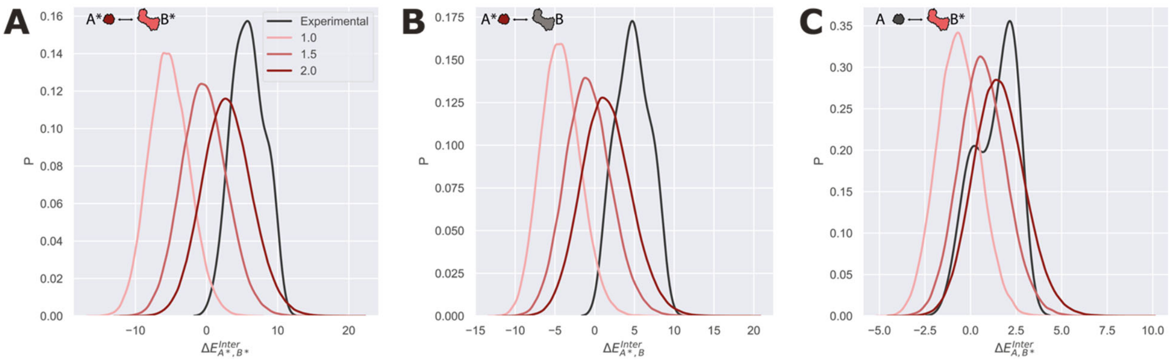

Fig. S3. Distributions of cognate and noncognate interaction scores of the mutants generated by direct sampling at various sampling temperatures and the experimentally determined mutants. (**A**) Cognate interactions $\Delta E_{Inter}(A^*,B^*)$. (**B**) $\Delta E_{Inter}(A^*,B)$. (**C**) $\Delta E_{Inter}(A,B^*)$. The different sampling temperatures are shown in shades of red, and the values for the experimentally determined mutants are shown in black.

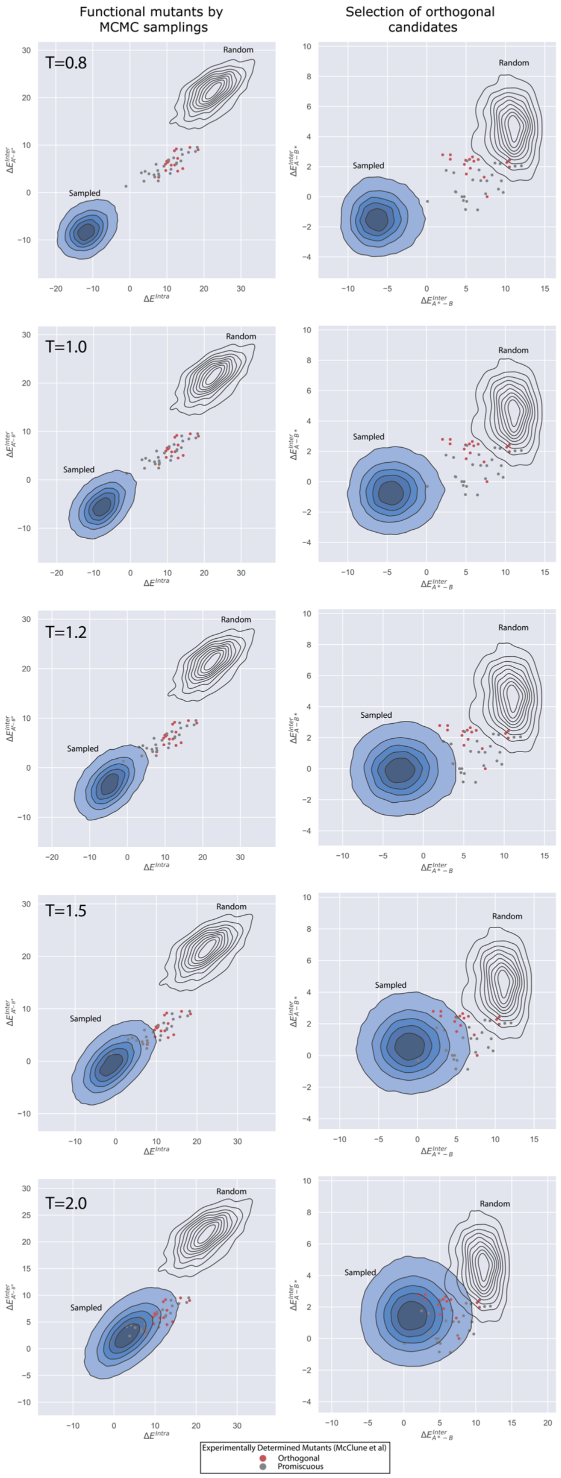

Fig. S4. The two stages of the design strategy for different sampling temperatures *T* in the MCMC model. Left: Generation of functional mutants by direct sampling. The two histograms show the density plots for 5×10^5 generated mutants, either by MCMC sampling (bottom left, shaded blue) or by random sampling (top right). Right: Selection of orthogonal candidates. The 5×10^5 mutants sampled by MCMC (blue shaded regions from left panels) were scored against their native partners to assess their likelihood of forming noncognate interactions. The 41 experimentally mutants reported by McClune *et al*. (*5*) are overlaid in all plots (red, orthogonal mutants; gray, promiscuous binding proteins).

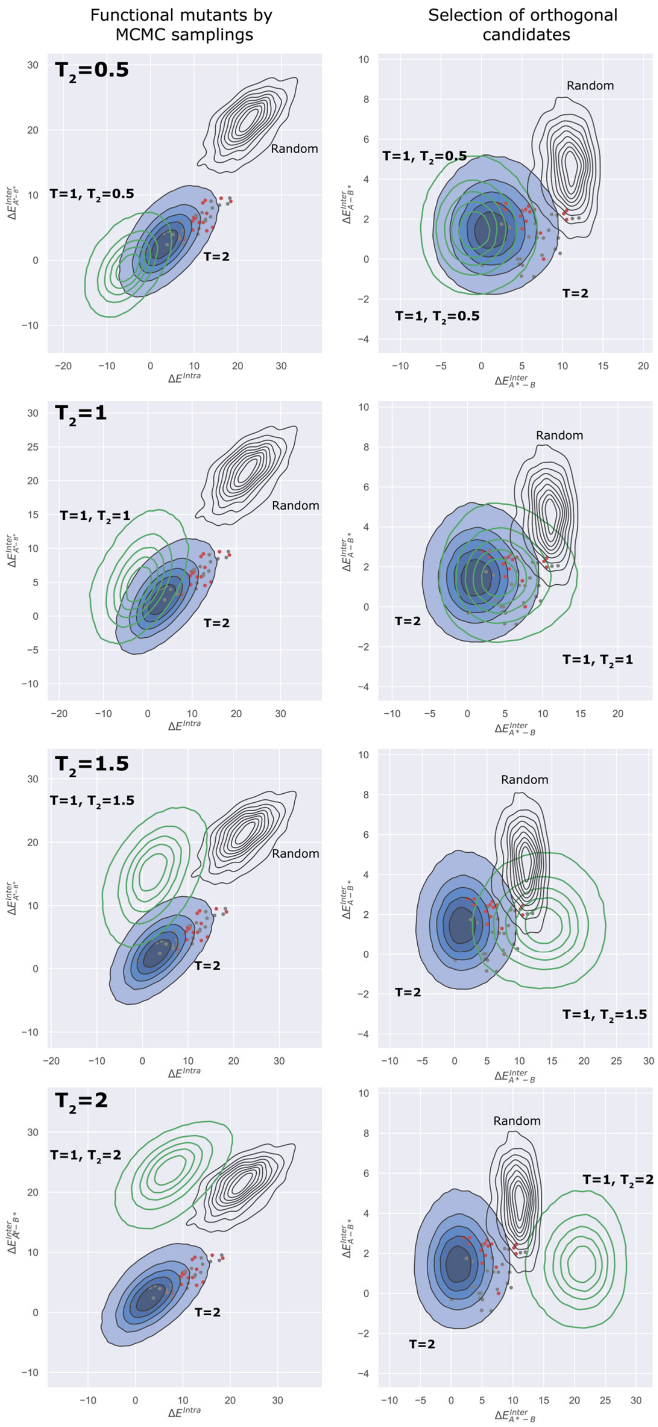

Fig. S5. Comparison of the energy distributions of the direct and conditional sampling strategies. The energy distributions of samples obtained with the conditional loss function [low E(A*,B*) and high E(A*,B), E(A,B*) at T = 1] at different weighting temperature (T_2) are shown in green contour are overlayed with samples obtained by direct sampling [E(A*,B*) at T = 2] shown in shaded blue contour and fully random samples shown as black contour. The direct sampling and random sample distributions correspond to the data presented in Fig. 3 and fig. S4. Dots represent the 41 experimentally tested mutants from McClune *et al*. (*5*) (red, orthogonal; gray, promiscuous).

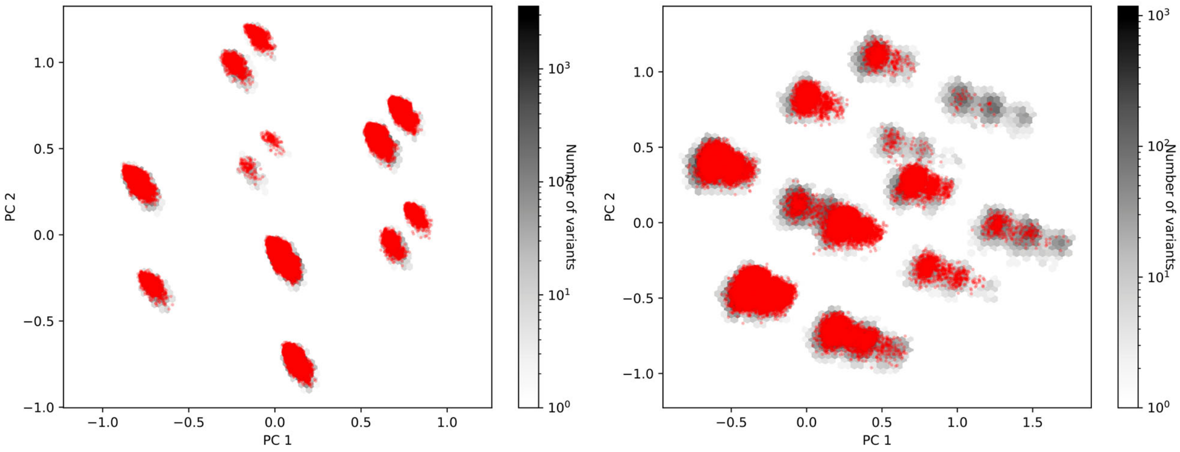

Fig. S6. Comparison of generated sequence repertoires in PCA space between direct sampling and selection of E(A*,B*) and conditional sampling with orthogonal selection. Left: PCA projections of the original direct sampling repertoire (gray dots) overlaid with the conditionally sampled repertoire (red dots). Right: PCA projections of the conditionally generated repertoire (gray dots), overlaid with the original direct sampling repertoire (red dots).

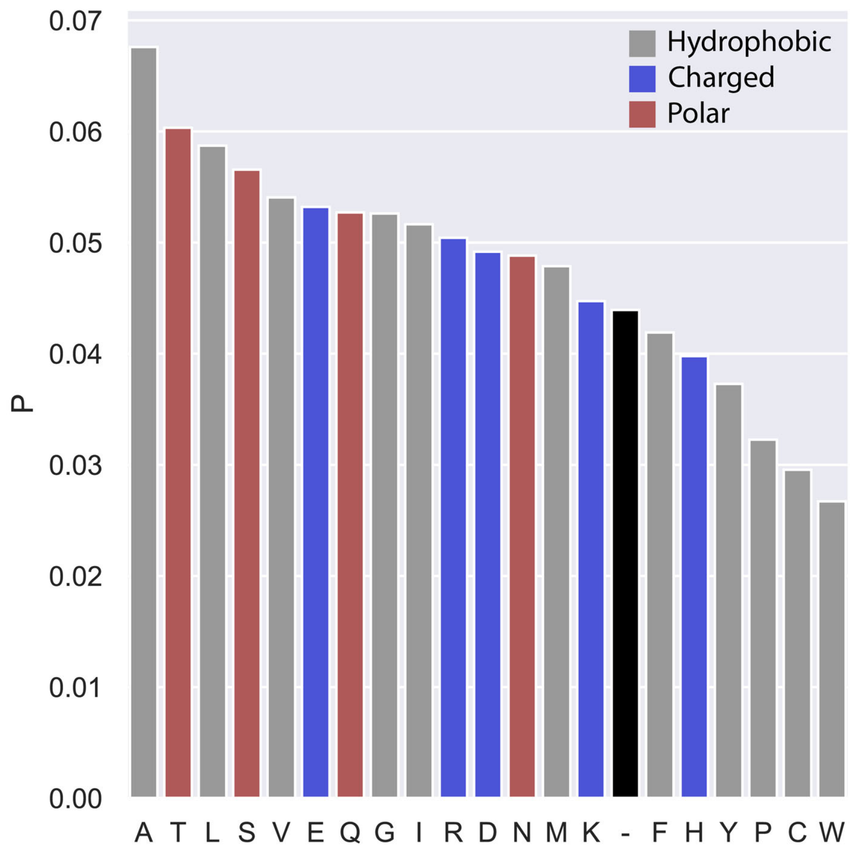

Fig. S7. Amino acid usage in the ~78,000 generated orthogonal variants. A dash (-) indicates a deletion (corresponding to gaps in the alignments).

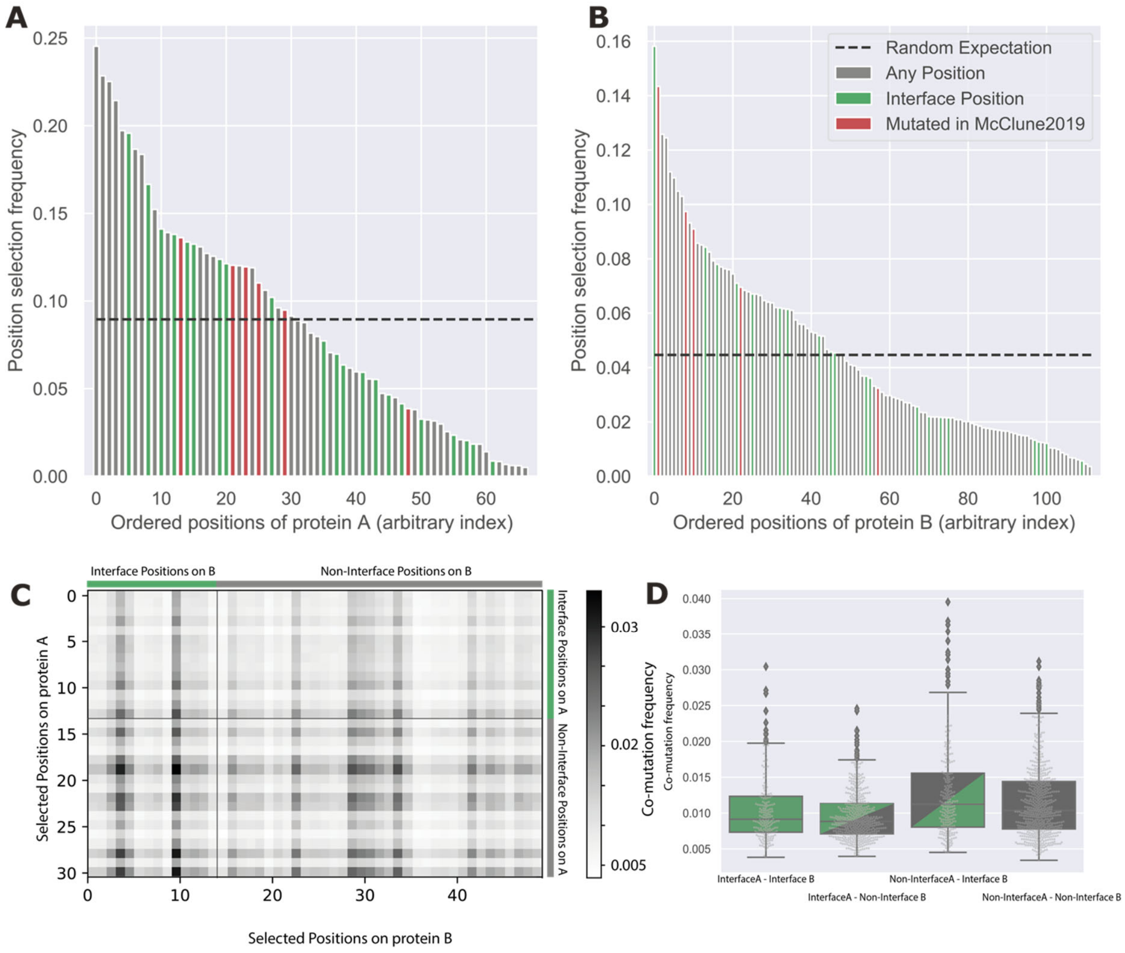

Fig. S8. Mutation frequencies and co-occurrences of intra- and inter-protein residue pairs. (**A** and **B**) Sorted mutation frequencies for each position on both proteins. The vertical axis shows the fraction of time that each position was mutated in the repertoire of the ~78,000 generated mutants. The horizontal dotted lines depict the selection frequency random expectations, which were computed by the hypergeometric null model. Panel A shows protein *A* (PhoQ); panel B shows protein *B* (PhoP). **(C)** Co-occurrence in the mutated repertoire of the positions above the selection threshold in (A) and (B). The positions were reordered to segregate the interface positions from the noninterface positions. (**D**) Comparison of the co-occurrence frequency of interface-interface and interface-noninterface positions between the two proteins. The box plot center lines show the medians, box limits show the upper and lower quartiles, and the whiskers show the 1.5 interquartile range. Outliers are depicted by diamonds. Individual data points are overlaid as gray dots.

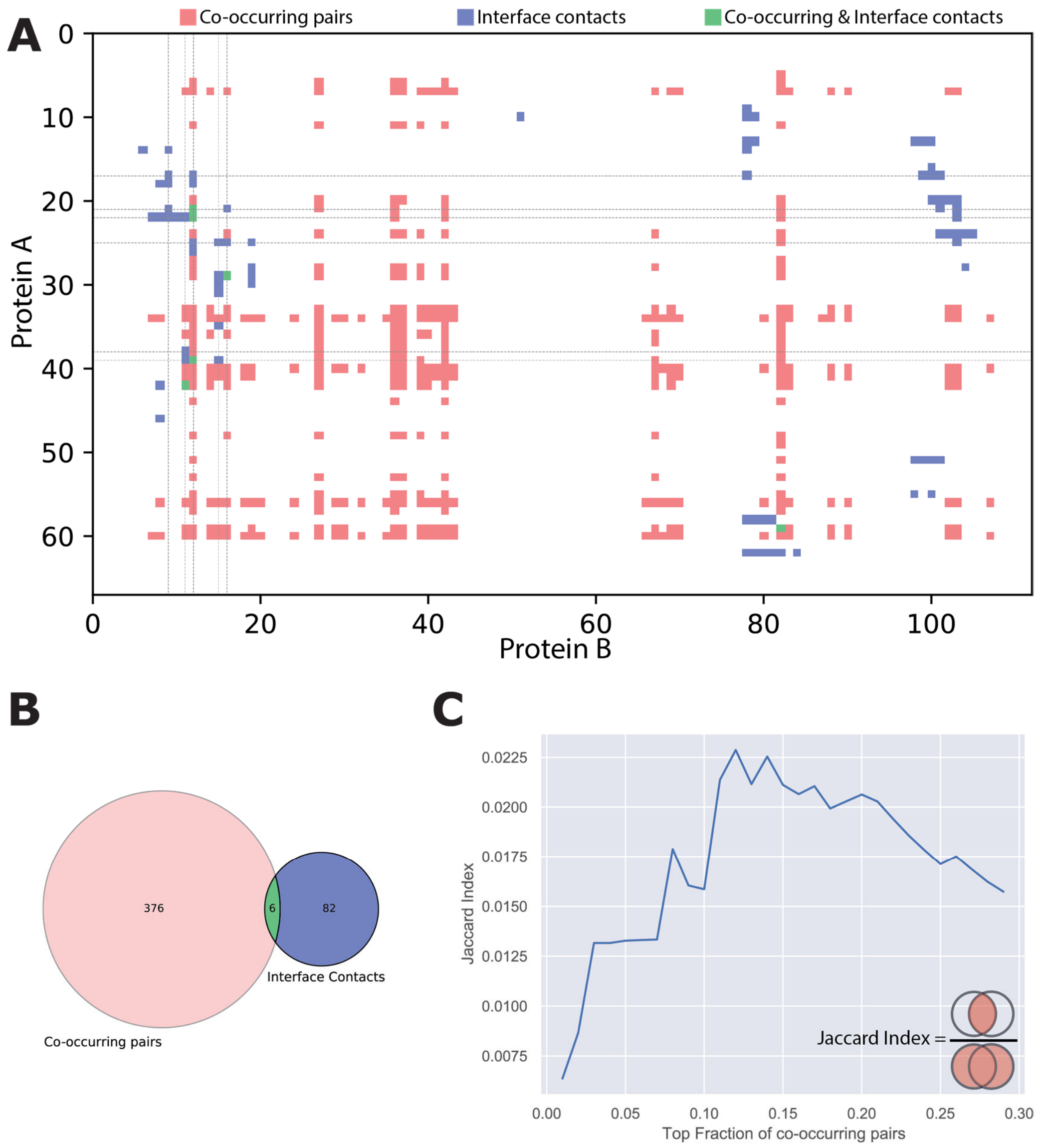

Fig. S9. Co-occurrence analysis in the repertoire generated by direct sampling and orthogonal selection. (**A**) Interface contact map depicting the interface contact occurring in the structural model (blue), pairs of mutations co-mutated in >5% of the generated mutants (red), and the intersection of both sets (green). Dotted line indicates the 11 mutated positions reported by McClune *et al*. (*5*). (**B**) Venn diagram showing the set intersection of the interface contacts and the 5% most co-occurring mutation pairs. (**C**) Jaccard Index of the interface contacts and the top co-occurring mutations pairs as a function of the fraction of top co-occurring mutated pairs.

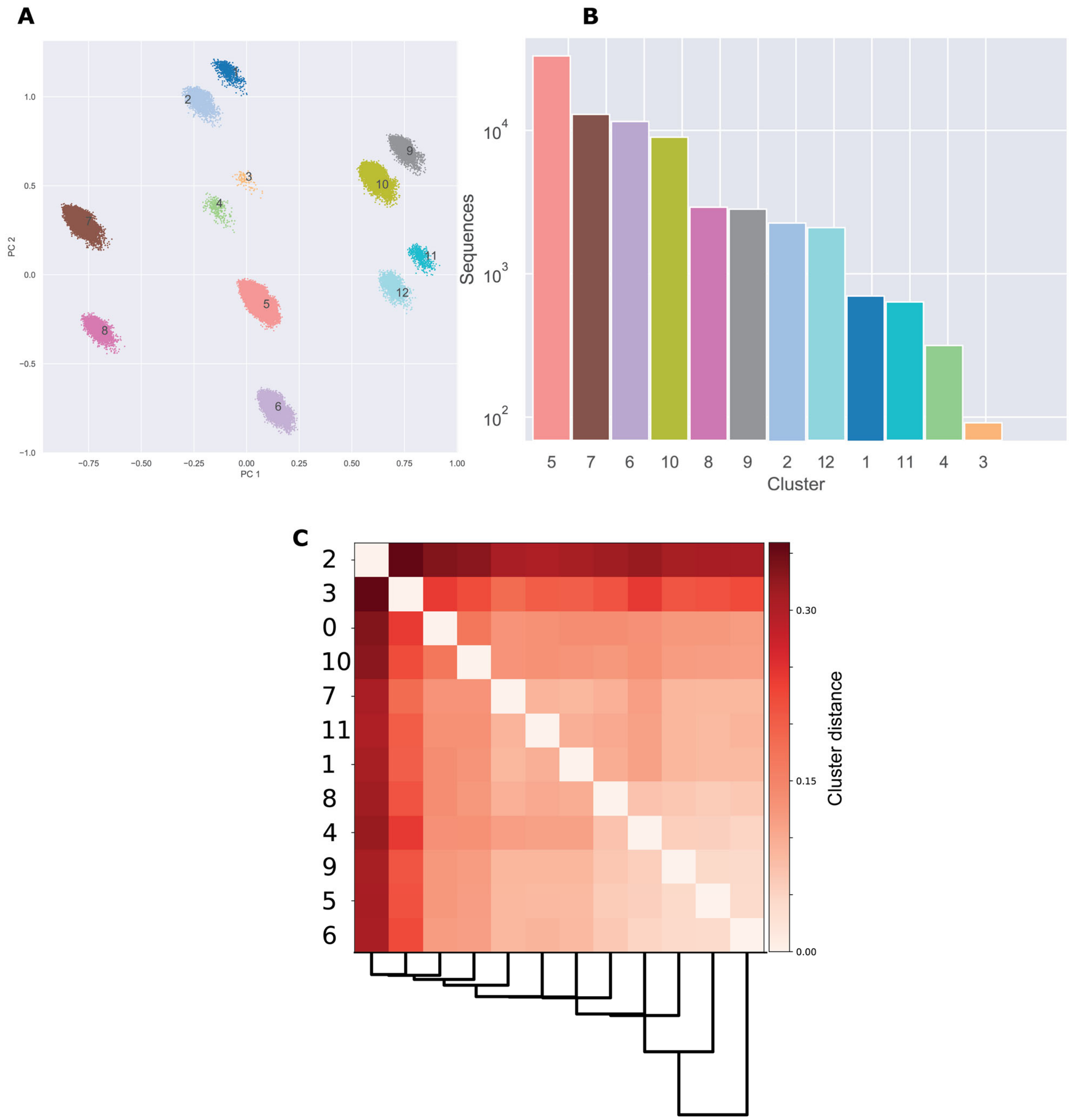

Fig. S10. Clustering of the set of mutants generated by direct sampling and orthogonal selection. (A) Identification of the 12 determined clusters in the space spanned by the two first PCs. (**B**) The number of mutant sequences in each cluster. (**C**) Similarity of the clusters as determined by hierarchical clustering. Cluster distances were computed as the Euclidean distance between the mutation profiles of the mutants in each pair of clusters (see figs. S11 and S12). Hierarchical clustering was performed with the single-linkage method.

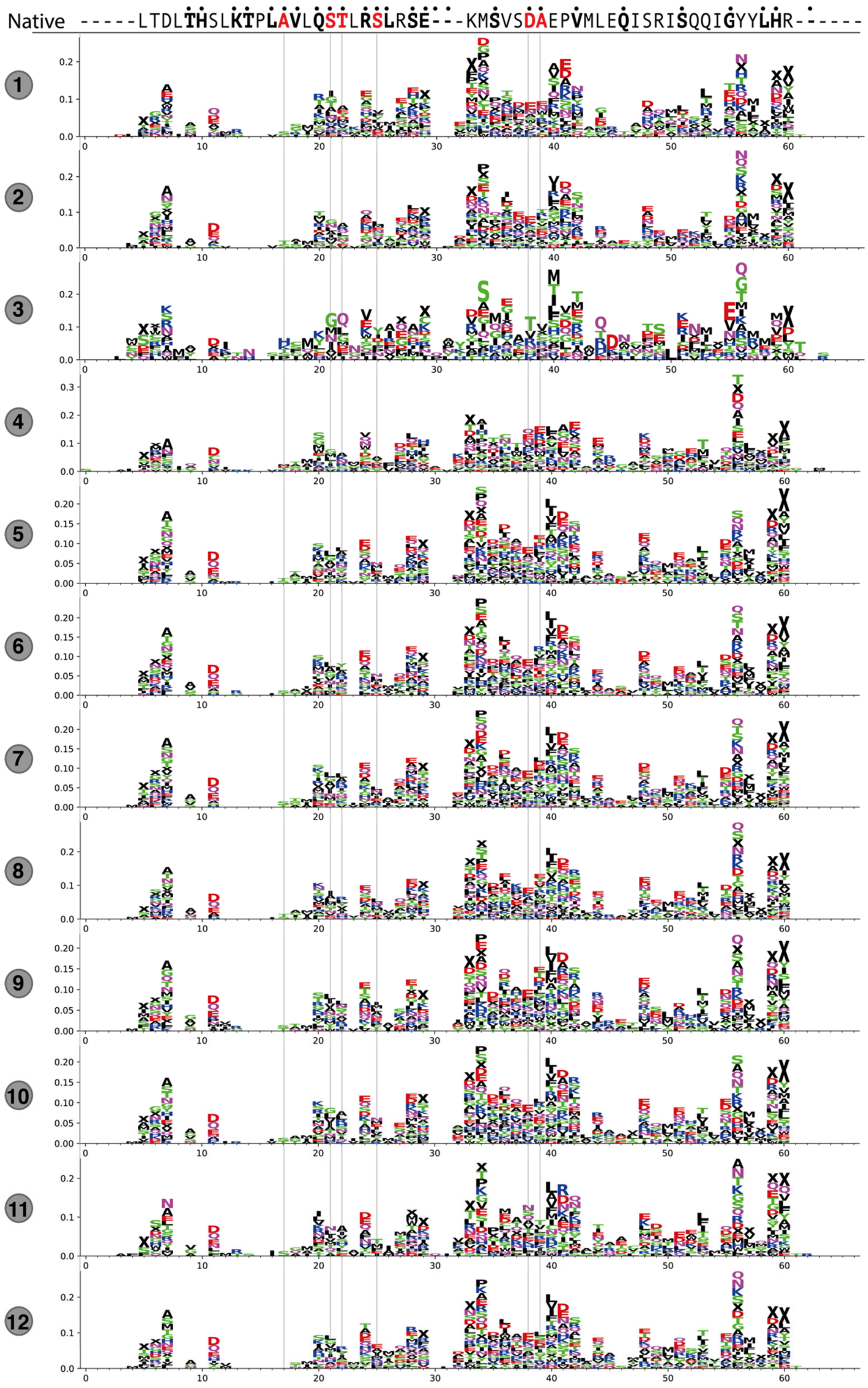

Fig. S11. Sequence logos for the HK domains of the 12 identified clusters. The logos depict normalized amino acid enrichment at each alignment position and were computed with the Logomaker library. The top sequence shows the native PhoQ sequence in the alignment. The six mutated positions reported by McClune *et al*. (*5*) are highlighted in red. Interface positions are denoted by a black dot on bold letters. For visual clarity, only mutations with a frequency >0.5% are shown.

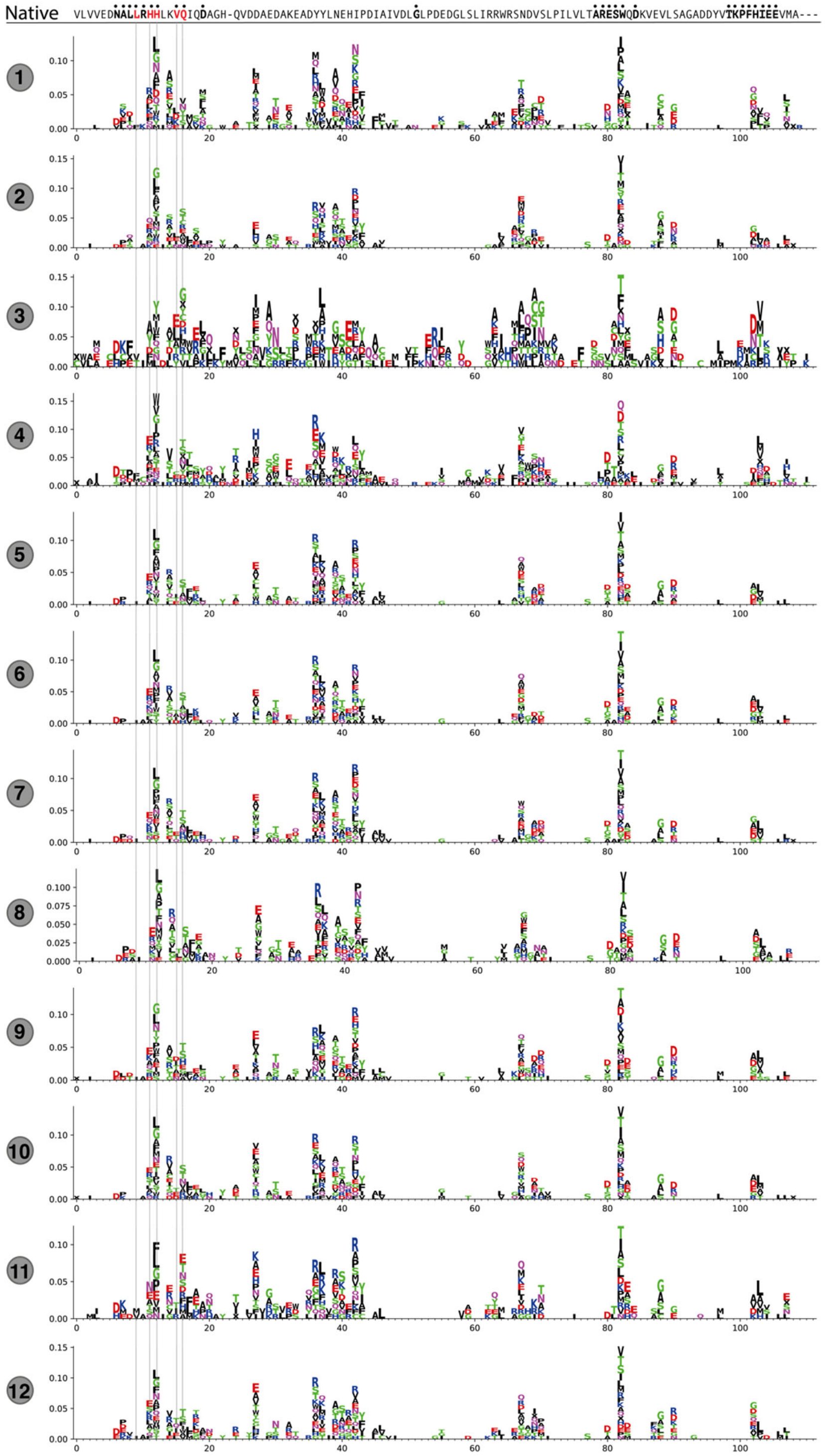

Fig. S12. Sequence logos for the RR domains of the 12 identified clusters. The logos depict the normalized amino acid enrichment at each alignment position and were computed with the Logomaker library. The top sequence shows the native PhoP sequence in the alignment. The five mutated positions reported by McClune *et al*. (*5*) are highlighted in red. Interface positions are denoted by a black dot on bold letters. For visual clarity, only mutations with a frequency >0.5% are shown.

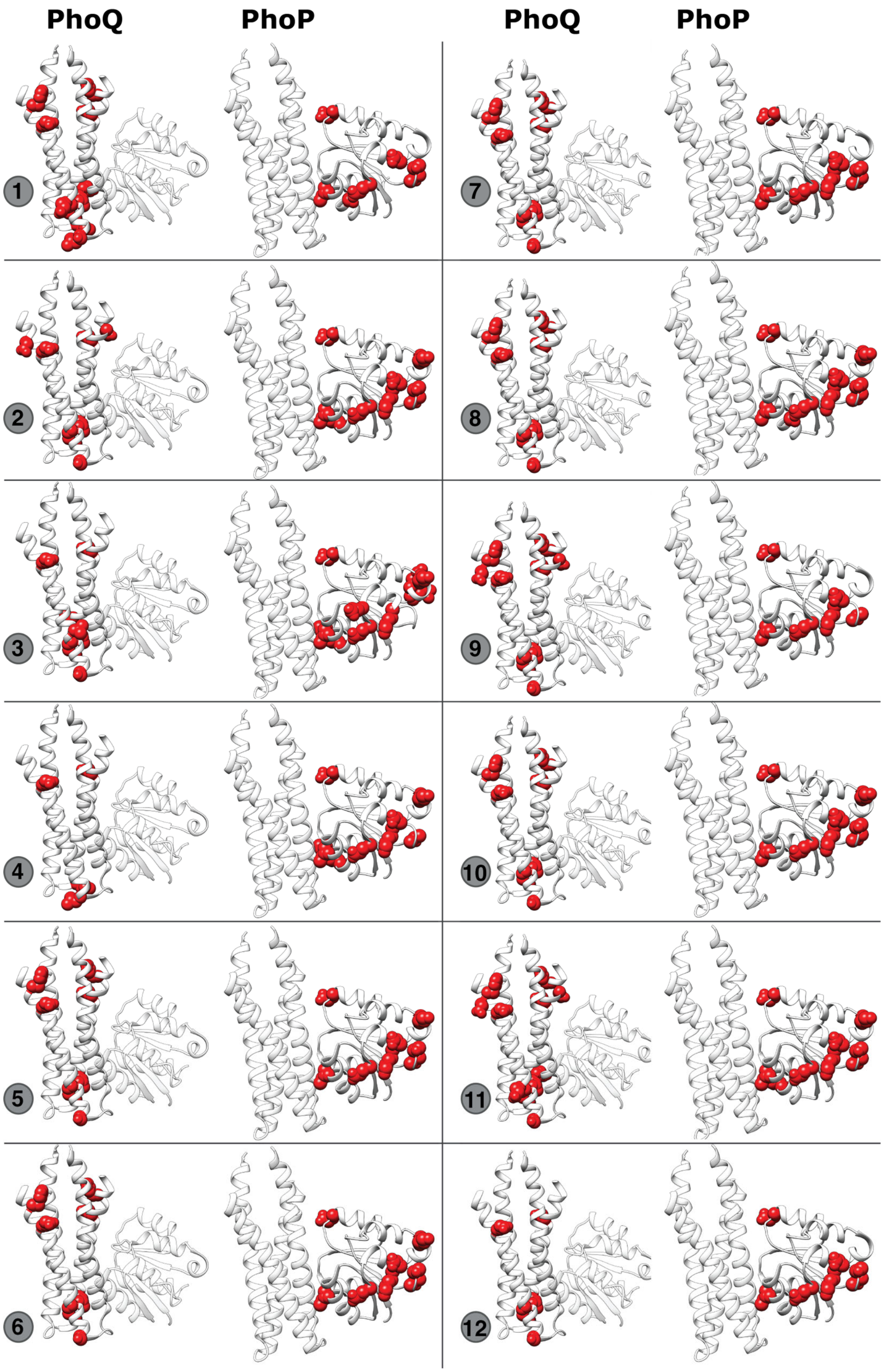

Fig. S13. Structural positions of the most mutated residues in the 12 identified mutant clusters. Cluster numbers are shown in gray circles. For each cluster, PhoQ is on the left and PhoP is on the right. For mutants of protein *A* (PhoQ homolog), the residue positions mutated in >10% of mutants in each cluster are highlighted as red spheres. For mutants of protein *B* (PhoP homolog), the residue positions mutated in >20% of mutants in each cluster are highlighted as red spheres.

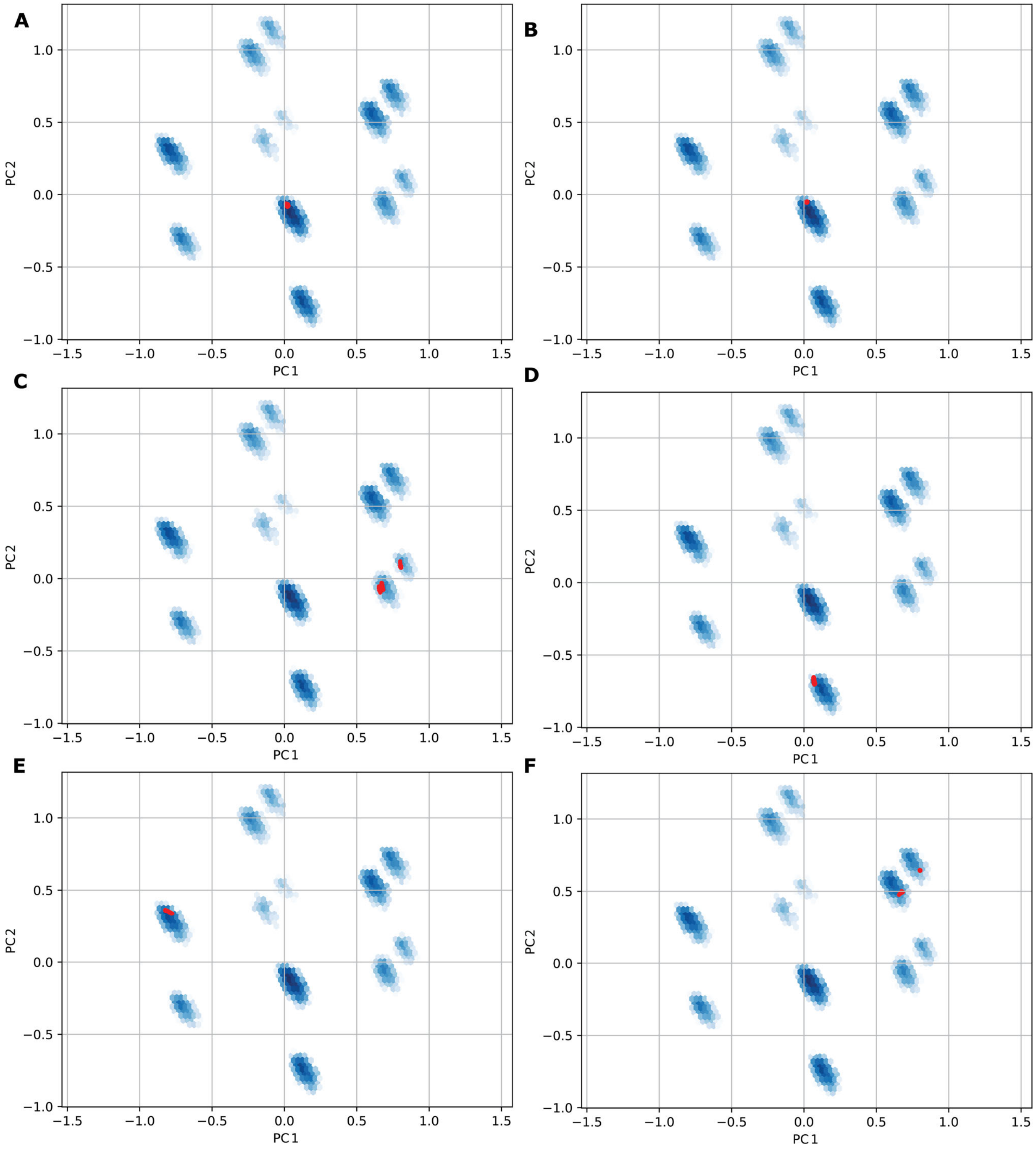

Fig. S14. Projection of position-constrained mutants generated by direct sampling and orthogonal selection onto the 12 clusters identified by PCA. (**A**) Mutants generated by only mutating the 11 positions as determined by McClune *et al*. (*5*). (**B**) A prototypical result of mutants generated by randomly constraining 11 positions among the 176. (**C** to **F**) Projections of variants generated with 11 fixed positions, which include positions often mutated in the base repertoire (see figs. S11 and S12). In each graph, the 12 clusters generated by direct sampling and orthogonal selection of the 78,000 mutant repertoire are shown in blue, with the constrained mutants displayed as red dots.

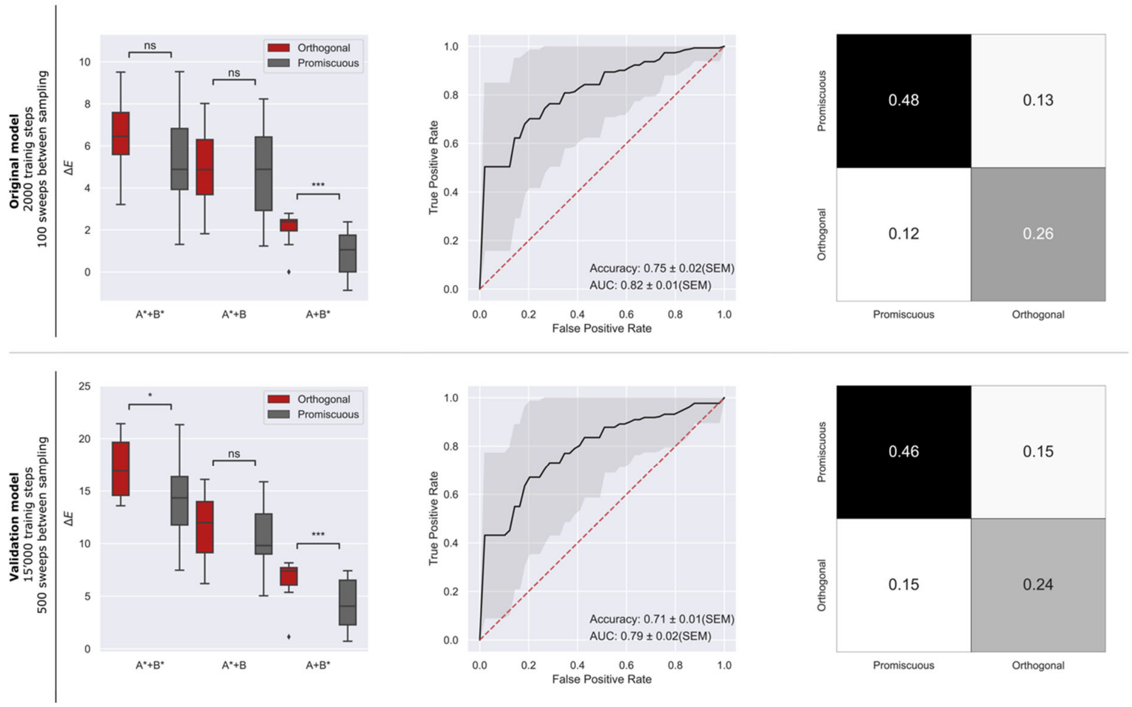

Fig. S15. Comparison of the main results using the normal and extended training regimes. Left: Scoring of the 41 experimentally determined mutants (16 orthogonal pairs and 25 promiscuous). All scores were normalized to the native scores. Statistical significance tests were computed by two-sided Mann–Whitney tests with Bonferroni correction (***$P < 0.001$). The box plot center lines show the medians, box limits show the upper and lower quartiles, and the whiskers show the 1.5 interquartile range. Outliers are depicted by diamonds. Middle: Receiver operator characteristic (ROC) curve of the linear classifier using the statistical scores as input features. The black solid curve shows the average ROC curve. The shaded area corresponds to ±1 SD computed over 100 randomized cross-validations. Right: Confusion matrix of the linear classifier using the statistical scores as input features. Each entry represents the total fraction of mutants in a reference group (Columns: ground truth: promiscuous or orthogonal) predicted to be in either of the two groups (Rows: predicted groups: promiscuous or orthogonal). The confusion matrix was averaged over 100 randomized cross-validations. Top: Original model used in this study (2000 training steps, 100 sweeps between samples). Bottom: Validation model (15,000 training steps, 500 sweeps between samples).

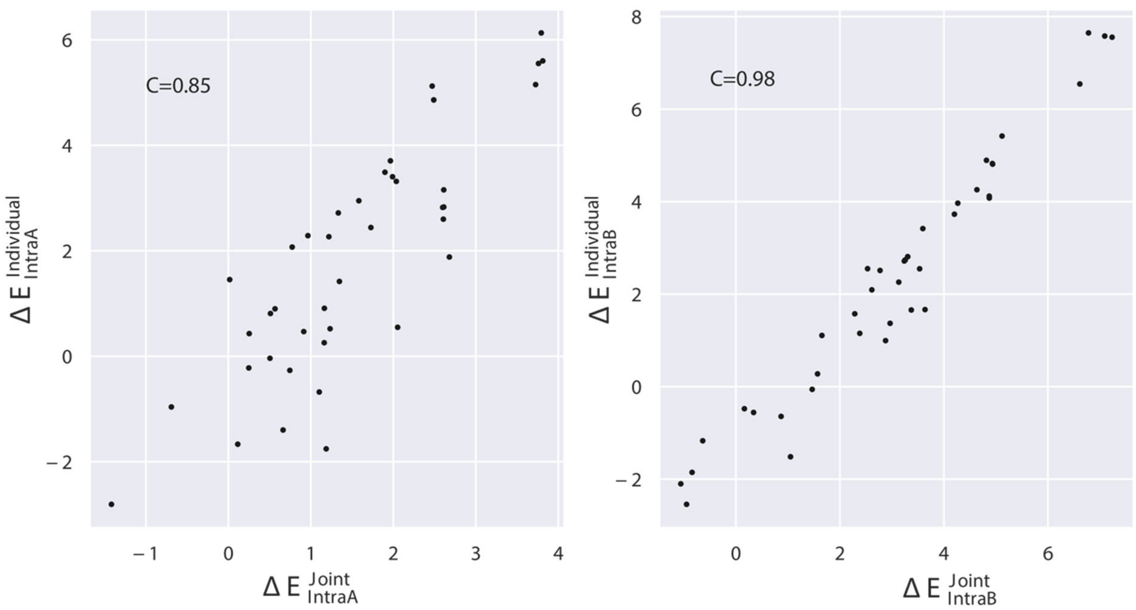

Fig. S16. Comparison of the statistical energies E_{IntraA} and E_{IntraB} for the full and independent model. Comparison of the statistical energies E_{IntraA} and E_{IntraB} of the 41 experimentally determined variants, computed with the full model used in this study and two independent models trained on the PhoQ and PhoP sequences independently. Left: E_{IntraA}. Right: E_{IntraB}. C, Pearson correlation coefficient.
